# Performance characteristics of five immunoassays for SARS-CoV-2: a head-to-head benchmark comparison

**DOI:** 10.1016/S1473-3099(20)30634-4

**Published:** 2020-12

**Authors:** The National SARS-CoV-2 Serology Assay Evaluation Group, Mark Ainsworth, Monique Andersson, Kathryn Auckland, J Kenneth Baillie, Eleanor Barnes, Sally Beer, Amy Beveridge, Sagida Bibi, Luke Blackwell, Martyna Borak, Abbie Bown, Tim Brooks, Nicola A Burgess-Brown, Susana Camara, Matthew Catton, Kevin K. Chau, Thomas Christott, Elizabeth Clutterbuck, Jesse Coker, Richard J Cornall, Stuart Cox, David Crawford-Jones, Derrick W Crook, Silvia D'Arcangelo, Wanwisa Dejnirattsai, Julie M M Dequaire, Stavros Dimitriadis, Kate E Dingle, George Doherty, Christina Dold, Tao Dong, Susanna J Dunachie, Daniel Ebner, Marc Emmenegger, Alexis Espinosa, David W Eyre, Rory Fairhead, Shayan Fassih, Conor Feehily, Sally Felle, Alejandra Fernandez-Cid, Maria Fernandez Mendoza, Thomas H Foord, Thomas Fordwoh, Deborah Fox McKee, John Frater, Veronica Gallardo Sanchez, Nick Gent, Dominique Georgiou, Christopher J Groves, Bassam Hallis, Peter M Hammond, Stephanie B. Hatch, Heli J Harvala, Jennifer Hill, Sarah J Hoosdally, Bryn Horsington, Alison Howarth, Tim James, Katie Jeffery, Elizabeth Jones, Anita Justice, Fredrik Karpe, James Kavanagh, David S Kim, Richard Kirton, Paul Klenerman, Julian C Knight, Leonidas Koukouflis, Andrew Kwok, Ullrich Leuschner, Robert Levin, Aline Linder, Teresa Lockett, Sheila F Lumley, Spyridoula Marinou, Brian D Marsden, Jose Martinez, Lucas Martins Ferreira, Lara Mason, Philippa C Matthews, Alexander J Mentzer, Alexander Mobbs, Juthathip Mongkolsapaya, Jordan Morrow, Shubhashish M M Mukhopadhyay, Matthew J Neville, Sarah Oakley, Marta Oliveira, Ashley Otter, Kevin Paddon, Jordan Pascoe, Yanchun Peng, Elena Perez, Prem K Perumal, Timothy E A Peto, Hayleah Pickford, Rutger J Ploeg, Andrew J Pollard, Anastasia Richardson, Thomas G Ritter, David J Roberts, Gillian Rodger, Christine S Rollier, Cathy Rowe, Justine K Rudkin, Gavin Screaton, Malcolm G Semple, Alex Sienkiewicz, Laura Silva-Reyes, Donal T Skelly, Alberto Sobrino Diaz, Lizzie Stafford, Lisa Stockdale, Nicole Stoesser, Teresa Street, David I Stuart, Angela Sweed, Adan Taylor, Hannah Thraves, Hoi P Tsang, Marije K Verheul, Richard Vipond, Timothy M Walker, Susan Wareing, Yolanda Warren, Charlie Wells, Clare Wilson, Kate Withycombe, Rebecca K Young

## Abstract

**Background:**

Severe acute respiratory syndrome coronavirus 2 (SARS-CoV-2) has caused a global pandemic in 2020. Testing is crucial for mitigating public health and economic effects. Serology is considered key to population-level surveillance and potentially individual-level risk assessment. However, immunoassay performance has not been compared on large, identical sample sets. We aimed to investigate the performance of four high-throughput commercial SARS-CoV-2 antibody immunoassays and a novel 384-well ELISA.

**Methods:**

We did a head-to-head assessment of SARS-CoV-2 IgG assay (Abbott, Chicago, IL, USA), LIAISON SARS-CoV-2 S1/S2 IgG assay (DiaSorin, Saluggia, Italy), Elecsys Anti-SARS-CoV-2 assay (Roche, Basel, Switzerland), SARS-CoV-2 Total assay (Siemens, Munich, Germany), and a novel 384-well ELISA (the Oxford immunoassay). We derived sensitivity and specificity from 976 pre-pandemic blood samples (collected between Sept 4, 2014, and Oct 4, 2016) and 536 blood samples from patients with laboratory-confirmed SARS-CoV-2 infection, collected at least 20 days post symptom onset (collected between Feb 1, 2020, and May 31, 2020). Receiver operating characteristic (ROC) curves were used to assess assay thresholds.

**Findings:**

At the manufacturers' thresholds, for the Abbott assay sensitivity was 92·7% (95% CI 90·2–94·8) and specificity was 99·9% (99·4–100%); for the DiaSorin assay sensitivity was 96·2% (94·2–97·7) and specificity was 98·9% (98·0–99·4); for the Oxford immunoassay sensitivity was 99·1% (97·8–99·7) and specificity was 99·0% (98·1–99·5); for the Roche assay sensitivity was 97·2% (95·4–98·4) and specificity was 99·8% (99·3–100); and for the Siemens assay sensitivity was 98·1% (96·6–99·1) and specificity was 99·9% (99·4–100%). All assays achieved a sensitivity of at least 98% with thresholds optimised to achieve a specificity of at least 98% on samples taken 30 days or more post symptom onset.

**Interpretation:**

Four commercial, widely available assays and a scalable 384-well ELISA can be used for SARS-CoV-2 serological testing to achieve sensitivity and specificity of at least 98%. The Siemens assay and Oxford immunoassay achieved these metrics without further optimisation. This benchmark study in immunoassay assessment should enable refinements of testing strategies and the best use of serological testing resource to benefit individuals and population health.

**Funding:**

Public Health England and UK National Institute for Health Research.

## Introduction

Severe acute respiratory syndrome coronavirus 2 (SARS-CoV-2) has emerged as a novel human pathogen, causing a global pandemic in 2020, with more than 25 million confirmed infections and more than 840 000 deaths to date.^1^ Testing and case ascertainment have been crucial to controlling virus transmission and in developing public health and political strategies to mitigate the effect of this pathogen.

SARS-CoV-2 testing broadly takes two forms: first, direct detection of the virus in respiratory samples with real-time RT-PCR; and second, by using serology to investigate the presence of antibodies.^2^ Immunoassays detect either specific types of antibody (eg, IgM or IgG) or total antibody. SARS-CoV-2 antibodies typically start to appear at least 5–7 days post infection^3^ and are therefore an unreliable marker for early acute infection. The degree and duration of immunity that antibodies confer are unclear. A prominent use for serological testing has therefore been at a population level, for informing the extent of population exposure. Other uses include assessing risk of infection at an individual level and to support research and development (eg, quantifying antibody responses in vaccine trials).^4^

Several manufacturers have developed immunoassays compatible with global laboratory infrastructures, enabling widespread testing of hundreds to thousands of samples per day. However, the scale-up required for regular population-wide testing (eg, every few weeks or months) might exceed the capacity of commercial platforms. To date, few thorough, direct assessments of immunoassay performance on large sample sets have been done, and governments, regulators, and clinical laboratories have had to balance the urgent need to facilitate the demand for serological testing with the few data available on assay performance, leading to a relaxation of typical assessment criteria (eg, the US Food and Drug Administration's [FDA] Emergency Use Authorization programme).^4^

**Research in context**

**Evidence before this study**

Substantial global interest exists in the use of serology to enable population-level surveillance of severe acute respiratory syndrome coronavirus 2 (SARS-CoV-2) infection and to inform individual-level management and risk stratification; however, it is unclear which widely available SARS-CoV-2 serological immunoassays perform to the standards required to meet these needs. The most widely used commercial assays that have obtained regulatory approvals for emergency use in the USA and the EU include the SARS-CoV-2 IgG assay (Abbott, Chicago, IL, USA), LIAISON SARS-CoV-2 S1/S2 IgG assay (DiaSorin, Saluggia, Italy), Elecsys Anti-SARS-CoV-2 assay (Roche, Basel, Switzerland), and the anti-SARS-COV-2 ELISA (IgG; EUROIMMUN, Lübeck, Germany). However, the sensitivity of the EUROIMMUN assay has been reported as being approximately 90%. We therefore replaced this with the SARS-CoV-2 Total assay (Siemens, Munich, Germany) in a head-to-head comparison. In addition to reviewing performance assessments undertaken by each manufacturer, we searched PubMed, BioRxiv, and MedRxiv up to May 31, 2020, using the following search terms: (SARS-CoV-2) AND ([ELISA] OR [EIA] OR [CLIA] OR [FIA] OR [IFA] OR [IgG]). Details and expanded PubMed search terms are given in the appendix (pp 7, 8). We also reviewed any investigations of relevant assays by Public Health England (PHE) up to May 31, 2020. Of 423 studies assessed, full-text reviews were done for 124 articles. Eight studies provided data on sensitivity and specificity for the Abbott (four studies) or DiaSorin (five) assays; PHE investigations were undertaken for the Abbott and Roche assays. For the Abbott assay, sensitivity ranged from 93·4% to 100% for samples taken at least 14 days post symptom onset (70 to 680 samples) and specificity from 95·1% to 100% (nine to 1020); for the DiaSorin assay, we found a single sensitivity estimate of 94·4% on samples taken at least 14 days post symptom onset (18 samples) and specificity ranged from 94·9% to 100% (69 to 1140 samples). For the Roche assay, we found a single sensitivity estimate of 87·0% on samples taken at least 14 days post symptom onset (n=77) and a single specificity estimate of 100% (n=472). No published studies assessed all four commercial platforms using the same sample sets.

**Added value of this study**

In this study on a large, single sample set of more than 1500 samples, we compared four commercial SARS-CoV-2 immunoassays (Abbott, DiaSorin, Roche, and Siemens assays) with a global installed base, and a novel 384-well ELISA (the Oxford immunoassay) that could scale to be used for population-level surveillance. The Siemens assay and Oxford immunoassay achieved a sensitivity and specificity of at least 98% without optimisation (lower 95% CI ≥96%). The other assays could achieve sensitivities and specificities of at least 98% with assay threshold adjustment or deployment on samples taken at least 30 days after symptom onset. However, differences observed in assay performance translate into thousands of additional incorrect diagnoses between the worst and best platforms if millions of tests are done in large populations. For example, at a 10% seroprevalence, the Siemens assay would generate an estimated 2800 total errors per million tests, versus the DiaSorin assay at a rate of 13 700 total errors per million tests.

**Implications of all the available evidence**

Understanding the comparative performance of immunoassays is crucial to the development of appropriate individual and population-level SARS-CoV-2 testing strategies. We showed that although precise performance metrics varied between immunoassay platforms, all assays that we assessed could be usefully deployed with careful consideration of use case, assay thresholds, and by considering symptom-to-sample timings, thus optimising the use of available serological testing resource and enabling the most widespread rollout. We also showed that a novel 384-well format ELISA would potentially be scalable for population-level testing of hundreds of thousands of samples.

To facilitate national public health and health-care providers in choosing platforms that are appropriate for SARS-CoV-2 serological testing, we aimed to directly assess the performance of four commercial SARS-CoV-2 antibody immunoassays, with the primary aim of identifying the sensitivity and specificity of each assay. We also assessed the same samples using a novel 384-well format ELISA that targeted antibodies against trimeric spike protein (the Oxford immunoassay).

## Methods

### Study design

To date, the UK has one of the few established standards for performance metrics for SARS-CoV-2 immunoassays internationally; no specific guidance exists on performance metrics from either the US FDA or the European Centre for Disease Prevention and Control. We established a blood (plasma and serum) sample collection including pre-pandemic samples from healthy individuals aged at least 18 years, collected between Sept 4, 2014, and Oct 4, 2016, as part of the Oxford Biobank,^5^ a population-based cohort of individuals in Oxfordshire, UK, who consented to participation in research studies, and samples from individuals aged at least 18 years with laboratory-confirmed SARS-CoV-2 infection from cohorts of patients admitted to hospital or surveillance on health-care workers (appendix p 9). These samples enabled us to assess the immunoassays in line with the UK Medicines and Healthcare products Regulatory Agency (MHRA) target product profile for enzyme immunoassays,^6^ requiring known negative samples to be taken more than 6 months before the known appearance of SARS-CoV-2 (ie, earlier than July 2019), and known positive samples to be collected from individuals with a previous positive SARS-CoV-2 RT-PCR from a nose or throat swab and at least 20 days post symptom onset (appendix pp 13, 14). The sample collection also enabled us to assess immunoassay performance on samples taken within 20 days after symptom onset as part of secondary analyses. In each case samples were de-duplicated by individual, and the latest sample from each individual was analysed. Two assays (SARS-CoV-2 IgG assay [Abbott, Chicago, IL, USA] and LIAISON SARS-CoV-2 S1/S2 IgG [DiaSorin, Saluggia, Italy]) were done in Oxford, UK (clinical biochemistry and microbiology laboratories, John Radcliffe Hospital), and two (Elecsys Anti-SARS-CoV-2 assay [Roche, Basel, Switzerland] and SARS-CoV-2 Total assay [Siemens, Munich, Germany]) at Public Health England (PHE) Porton Down.

Additionally, we analysed an eight-point 1:2 dilution series of three known high-volume plasma samples obtained from SARS-CoV-2-positive patients recruited by the UK National Health Service Blood and Transplant (NHSBT; RT-PCR positive, at least 20 days post symptom onset) with high (donor ID 10062), medium (10061), and low (10063) titre SARS-CoV-2 IgG antibodies as assessed by the SARS-CoV-2 IgG ELISA (EUROIMMUN, Lübeck, Germany; ratio values of 33·33, 4·34, and 2·50, respectively; ratio is the optical density of the sample divided by the optical density of the calibrator [ratio ≥1·1 is positive]), and one known negative control (BD001). Samples were analysed at minimum in triplicate on the four commercial immunoassays, and singly on the Oxford immunoassay. The NHSBT samples were correlated with national SARS-CoV-2 reagents developed by the UK's National Institute for Biological Standards and Control (NIBSC; 20/130 [single donor, high-titre antibody], 20/120 [single donor, relatively high-titre antibody], 20/122 [pool of five donor samples, mid-titre antibody], 20/124 [low S1, high-nucleocapsid protein antibody titre], 20/126 [low-titre antibody], 20/128 [negative control]^7^, ^8^) on the Oxford immunoassay (appendix p 10).

We drafted a protocol before implementing the study, which was shared with the manufacturers and the UK Department of Health and Social Care.

### Procedures

The installed base of analysers running the investigated assays is global, making their widespread deployment feasible. Two assays targeted the nucleocapsid protein (Abbott and Roche) and two had spike protein-based targets (DiaSorin and Siemens); two were IgG assays (Abbott and DiaSorin), and two were total antibody assays (Roche and Siemens). Manufacturers' sensitivity estimates ranged from 96·8% to 100% on samples taken at least 14 days post symptom onset or RT-PCR, and specificity estimates ranged from 98·5% to 99·8% on pre-pandemic samples (appendix p 14).^9^, ^10^, ^11^, ^12^, ^13^, ^14^, ^15^, ^16^, ^17^, ^18^

Assays were done in accordance with the manufacturers' instructions by trained laboratory staff in laboratories that were accredited by the UK Accreditation Service, on appropriate analysers, and with the specified controls and calibrants, using thresholds for calling positives and negatives set by the manufacturer for testing in the UK (appendix p 14), but also reporting quantitative values. Samples were assigned unique study barcodes for this study, and laboratory staff doing the assays did not have access to sample metadata before running the assays.

The Oxford immunoassay is an indirect ELISA, measuring serum IgG against trimeric spike protein,^19^ using a horseradish peroxidase-linked anti-human IgG antibody as the secondary. We implemented this assay as previously described,^20^ with minor modifications (appendix p 10). Readouts were measured as a fluorescent signal, normalised to standard units by calibrating to a dilution series of the NHSBT controls and a monoclonal antibody (CR3022), using a natural cubic spline-based regression model to enable across-plate and across-batch comparisons (appendix p 10). We derived thresholds for the Oxford immunoassay using an independent set of serum samples from 44 individuals with acute SARS-CoV-2 infection; 99 convalescent individuals post SARS-CoV-2 infection; one Middle East respiratory syndrome coronavirus (MERS-CoV) antiserum; 23 individuals with other respiratory virus infections; and 1205 pre-pandemic samples (appendix pp 10, 11). After at least 10 days post symptom onset, excluding the MERS-CoV antiserum (which was positive) but including the 23 samples from individuals with other viral infections, derivation sensitivity was 100% (120 of 120 [95% CI 97·0–100·0]) and specificity (at a normalised threshold of 8 million units) was 99·6% (1223 of 1228 [99·1–99·9).

The Oxford immunoassay runs batches of up to 12 384-well plates, each containing 3200 samples, over a period of 7 h to generate data on up to 3840 samples and controls per run. Samples and plates were handled using a Janus automated liquid handler (Perkin Elmer, Waltham, MA, USA), and plates were read with an EnVision 2104 multilabel plate reader (Perkin Elmer). The assay requires low input sample volumes of the order of 2 μL serum sample, subsequently diluted 1:25 in sample buffer (1% milk in phosphate-buffered saline with Tween); however, dead volume requirements of approximately 10 μL are required for adequate liquid handling function.

Sample aliquots of 0·5–2 mL were stored at −20°C before the study, and freeze-thaw cycles were minimised (fewer than five cycles). After thawing for processing, samples were spun for 10 min at 1844 g and the supernatant removed for subsequent work. No further freeze or thaw steps were taken during the study and samples were kept at 4°C for up to 7 days to complete all runs. For the Oxford immunoassay assessment, thawed samples were arrayed in 384-well plates and stored at −20°C with a single further thaw before processing.

### Outcomes and statistical analysis

Whereas sample numbers were partly decided by the availability of samples required to address the study aims, the sample size was prospectively calculated to provide 80% power to detect a lower 95% CI of at least 96·0% if the actual assay sensitivity was 98·3% (which required ≥460 positive samples) and 80% power to detect a lower 95% CI of at least 96·0% if the actual assay specificity was 97·7% (which required ≥901 negative samples). These metrics were in line with the manufacturers' performance characteristics.

The data were collated, cleaned, and locked before analysis. Sensitivity and specificity with exact binomial 95% CIs for each assay were compared with current UK MHRA guidance^6^ stipulating a requirement for sensitivity and specificity of at least 98%, with the lower bound of the 95% CI to be at least 96%. Analyses were carried out for samples for which results were available across all platforms as the primary analysis, and for all samples as part of secondary analyses. When more than one sample was available from the same individual, samples were de-duplicated by individual and only the last sample obtained was analysed, unless otherwise indicated.

Other secondary analyses were done; first, using receiver operator characteristic (ROC) curves to define trade-offs in assay sensitivity and specificity. Assay performance was also assessed according to the sampling timepoint (days post symptom onset and post RT-PCR test; at intervals of ≥14 days [per original protocol], ≥20 days [per MHRA], and ≥30 days). Evidence of differences in immunoassay sensitivity with respect to symptom severity (defined as asymptomatic, mild, severe, critical or death, in line with WHO criteria^21^) for the subgroup of samples for which this information was available was assessed for each immunoassay. Positive and negative predictive values at population prevalences of 5%, 10%, 20%, and 50% previous SARS-CoV-2 infection were modelled. The percentage of positive tests from SARS-CoV-2-positive individuals confirmed by RT-PCR over time and by serology platform was assessed; for this analysis all samples were included. Trajectories of antibody titres with respect to days post symptom onset were modelled by fitting spline curves (three to five knots; model selection based on the Akaike Information Criterion); for this analysis the first sample per patient was included. Statistical analyses and data visualisations were done in R (version 3.6.3) and Stata/IC (version 16.1). Analyses and results were overseen by an external review group.

For the DiaSorin assay, for which the manufacturer specifies repeat testing in the event that results fall within an equivocal zone (12·0 ≤ × <15·0 AU/mL), we pre-specified that we were unable to do repeat testing because of insufficient sample volumes available. These samples were excluded from the primary sensitivity and specificity calculations for the DiaSorin assay, but subsequently included in the ROC analyses. STARD and PRISMA checklists are included in the appendix (pp 37–40).

### Role of the funding source

The funder of the study had no role in study design, data collection, data analysis, data interpretation, or writing of the report. The corresponding author had full access to all the data in the study and had final responsibility for the decision to submit for publication.

## Results

**Figure 1:**
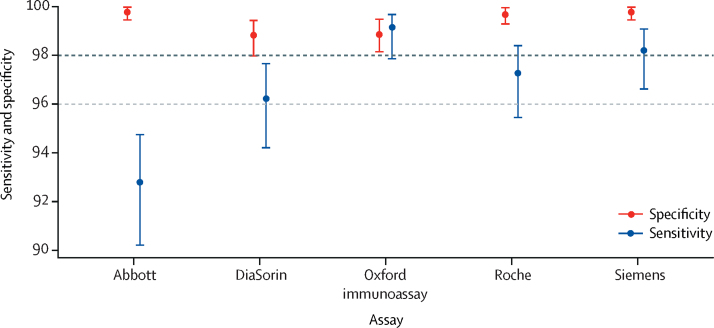
Sensitivity and specificity for each assay on samples taken ≥20 days post symptom onset in patients with laboratory-confirmed SARS-CoV-2 infection for positive samples, and >6 months before the first known COVID-19 cases for negative samples The UK MHRA performance target is shown (dark grey dashed line), including the required lower bound of the 95% CI (light grey dashed line) for sensitivity and specificity. Data are presented for 976 known-negative samples and 536 known-positive samples run on each assay. Equivocal results were excluded from the calculation of sensitivity and specificity for the DiaSorin assay (n=9). MHRA=UK Medicines and Healthcare products Regulatory Agency. Abbott=SARS-CoV-2 IgG assay (Abbott, Chicago, IL, USA). DiaSorin=LIAISON SARS-CoV-2 S1/S2 IgG assay (DiaSorin, Saluggia, Italy). Oxford immunoassay=a novel 384-well format ELISA (University of Oxford, Oxford, UK). Roche=Elecsys Anti-SARS-CoV-2 assay (Roche, Basel, Switzerland). SARS-CoV-2=severe acute respiratory syndrome coronavirus 2. Siemens=SARS-CoV-2 Total assay (Siemens, Munich, Germany).

**Figure 2:**
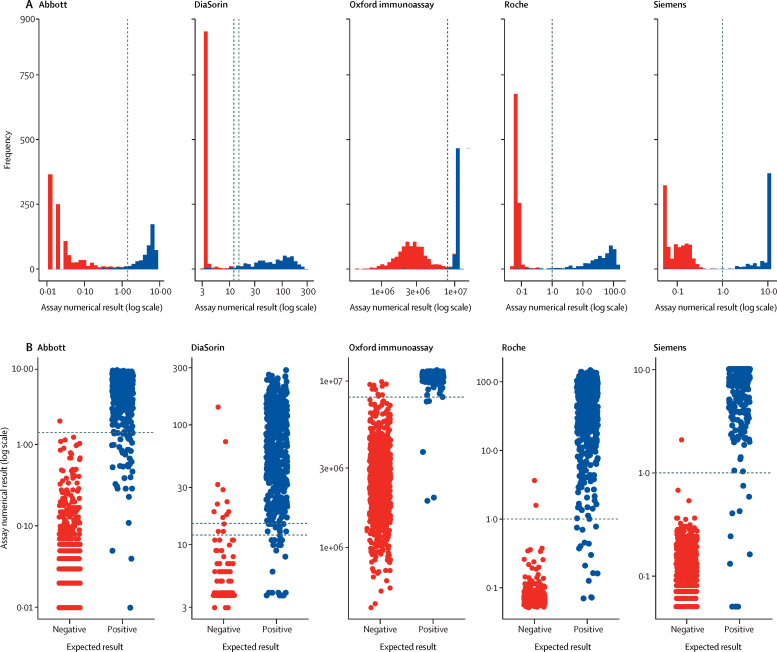
Distribution of numerical results obtained for each commercial assay Results are represented as (A) histograms, to enable assessment of the frequency of values, and (B) dotplots, to review scatter of values, especially around thresholds. Prespecified assay thresholds are shown as dashed lines. For the purposes of plotting values on a log scale, values of zero were set to the lowest non-zero value and results of greater or less than the largest or smallest values were truncated to the largest and smallest values. Data are presented for 976 known negative samples and 536 known positive samples run across all assays. Abbott=SARS-CoV-2 IgG assay (Abbott, Chicago, IL, USA). DiaSorin=LIAISON SARS-CoV-2 S1/S2 IgG assay (DiaSorin, Saluggia, Italy). Oxford immunoassay=a novel 384-well format ELISA (University of Oxford, Oxford, UK). Roche=Elecsys Anti-SARS-CoV-2 assay (Roche, Basel, Switzerland). SARS-CoV-2=severe acute respiratory syndrome coronavirus 2. Siemens=SARS-CoV-2 Total assay (Siemens, Munich, Germany).

From 1000 pre-pandemic samples (collected between Sept 4, 2014, and Oct 4, 2016), and 769 samples from 673 individuals with laboratory-confirmed SARS-CoV-2 infection (collected between Feb 1, and May 31, 2020), assay sensitivity and specificity were assessed on 976 known negative pre-pandemic samples and 536 samples from RT-PCR-confirmed COVID-19 patients, de-duplicated by individual for whom complete data across all platforms were available (appendix p 24). No samples failed testing on any of the four commercial platforms. We found 18 liquid handling failures on the Oxford immunoassay resulting in empty wells. Sensitivity and specificity of each of the assays are shown in the table and figure 1. Similar results were obtained analysing all available samples (appendix p 27; table). The clearest separation of known positive and known negative samples was shown for the Siemens assay (figure 2; appendix p 27 [all samples]).

**Table:** Sensitivity, specificity, and sample sizes for all groups and subgroups assessed

	**Specificity**						**Sensitivity**					
	Detected	Equivocal	Not detected	Not available	Total	Specificity (95% CI)	Detected	Equivocal	Not detected	Not available	Total	Sensitivity (95% CI)
**Samples with complete data available**												
Abbott	1	..	975	..	976	99·9% (99·4–100)	497	..	39	..	536	92·7% (90·2–94·8)
DiaSorin	11	2	963	..	976	98·9% (98·0–99·4)	509	7	20	..	536	96·2% (94·2–97·7)
Oxford immunoassay	10	..	966	..	976	99·0% (98·1–99·5)	531	..	5	..	536	99·1% (97·8–99·7)
Roche	2	..	974	..	976	99·8% (99·3–100)	521	..	15	..	536	97·2% (95·4–98·4)
Siemens	1	..	975	..	976	99·9% (99·4–100)	526	..	10	..	536	98·1% (96·6–99·1)
**Samples with complete data available (specificity set to ≥98%)**												
Abbott	19	..	957	..	976	98·1% (97·0–98·8)	523	..	13	..	536	97·6% (95·9–98·7)
DiaSorin	18	0	958	..	976	98·2% (97·1–98·9)	523	0	13	..	536	97·6% (95·9–98·7)
Oxford immunoassay	19	..	957	..	976	98·1% (97·0–98·8)	533	..	3	..	536	99·4% (98·4–99·9)
Roche	19	..	957	..	976	98·1% (97·0–98·8)	533	..	3	..	536	99·4% (98·4–99·9)
Siemens	15	..	961	..	976	98·5% (97·5–99·1)	530	..	6	..	536	98·9% (97·6–99·6)
**All samples**												
Abbott	1	..	994	..	995	99·9% (99·4–100)	500	..	40	..	540	92·6% (90·0–94·7)
DiaSorin	12	2	981	..	995	98·8% (97·9–99·4)	512	7	21	..	540	96·1% (94·0–97·5)
Oxford immunoassay	10	..	967	18	977	99·0% (98·1–99·5)	535	..	5	..	540	99·1% (97·9–99·7)
Roche	2	..	993	..	995	99·8% (99·3–100)	521	..	15	4	536	97·2% (95·4–98·4)
Siemens	1	..	993	1	994	99·9% (99·4–100)	526	..	10	4	536	98·1% (96·6–99·1)
**All samples (specificity set to ≥98%)**												
Abbott	19	..	976	..	995	98·1% (97·0–98·8)	527	0	13	..	540	97·6% (95·9–98·7)
DiaSorin	19	0	976	..	995	98·1% (97·0–98·8)	527	..	13	..	540	97·6% (95·9–98·7)
Oxford immunoassay	19	..	958	18	977	98·1% (97·0–98·8)	537	..	3	..	540	99·4% (98·4–99·9)
Roche	19	..	976	..	995	98·1% (97·0–98·8)	533	..	3	4	536	99·4% (98·4–99·9)
Siemens	15	..	979	1	994	98·5% (97·5–99·2)	530	..	6	4	536	98·9% (97·6–99·6)
**Samples with complete data available taken ≥14 days post symptom onset**												
Abbott	1	..	975	..	976	99·9% (99·4–100)	520	..	41	..	561	92·7% (90·2–94·7)
DiaSorin	11	2	963	..	976	98·9% (98·0–99·4)	529	8	24	..	561	95·7% (93·6–97·2)
Oxford immunoassay	10	..	966	..	976	99·0% (98·1–99·5)	554	..	7	..	561	98·8% (97·4–99·5)
Roche	2	..	974	..	976	99·8% (99·3–100)	543	..	18	..	561	96·8% (95·0–98·1)
Siemens	1	..	975	..	976	99·9% (99·4–100)	548	..	13	..	561	97·7% (96·1–98·8)
**Samples with complete data available taken ≥14 days post symptom onset (specificity set to ≥98%)**												
Abbott	19	..	957	..	976	98·1% (97·0–98·8)	546	..	15	..	561	97·3% (95·6–98·5)
DiaSorin	18	0	958	..	976	98·2% (97·1–98·9)	544	0	17	..	561	97·0% (95·2–98·2)
Oxford immunoassay	19	..	957	..	976	98·1% (97·0–98·8)	557	..	4	..	561	99·3% (98·2–99·8)
Roche	19	..	957	..	976	98·1% (97·0–98·8)	556	..	5	..	561	99·1% (97·9–99·7)
Siemens	15	..	961	..	976	98·5% (97·5–99·1)	554	..	7	..	561	98·8% (97·4–99·5)
**Samples with complete data available taken ≥30 days post symptom onset**												
Abbott	1	..	975	..	976	99·9% (99·4–100)	458	..	32	..	490	93·5% (90·9–95·5)
DiaSorin	11	2	963	..	976	98·9% (98·0–99·4)	468	6	16	..	490	96·7% (94·7–98·1)
Oxford immunoassay	10	..	966	..	976	99·0% (98·1–99·5)	487	..	3	..	490	99·4% (98·2–99·9)
Roche	2	..	974	..	976	99·8% (99·3–100)	481	..	9	..	490	98·2% (96·5–99·2)
Siemens	1	..	975	..	976	99·9% (99·4–100)	482	..	8	..	490	98·4% (96·8–99·3)
**Samples with complete data available taken ≥30 days post symptom onset (specificity set to ≥98%)**												
Abbott	19	..	957	..	976	98·1% (97·0–98·8)	482	..	8	..	490	98·4% (96·8–99·3)
DiaSorin	18	0	958	..	976	98·2% (97·1–98·9)	481	0	9	..	490	98·2% (96·5–99·2)
Oxford immunoassay	19	..	957	..	976	98·1% (97·0–98·8)	488	..	2	..	490	99·6% (98·5–100)
Roche	19	..	957	..	976	98·1% (97·0–98·8)	488	..	2	..	490	99·6% (98·5–100)
Siemens	15	..	961	..	976	98·5% (97·5–99·1)	485	..	5	..	490	99·0% (97·6–99·7)

Specificity was assessed using the known negative pre-pandemic samples, and sensitivity using the known positive samples from patients who had previously had laboratory-confirmed SARS–CoV–2 infection. Abbott=SARS-CoV-2 IgG assay (Abbott, Chicago, IL, USA). DiaSorin=LIAISON SARS-CoV-2 S1/S2 IgG assay (DiaSorin, Saluggia, Italy). Oxford immunoassay=a novel 384-well format ELISA (University of Oxford, Oxford, UK). Roche=Elecsys Anti-SARS-CoV-2 assay (Roche, Basel, Switzerland). SARS-CoV-2=severe acute respiratory syndrome coronavirus 2. Siemens=SARS-CoV-2 Total assay (Siemens, Munich, Germany).

**Figure 3:**
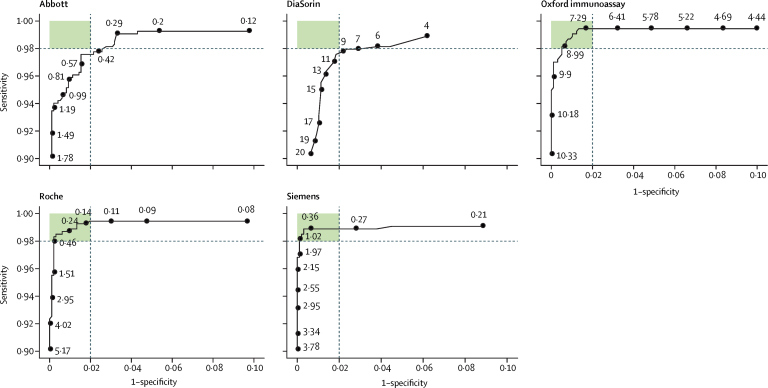
ROC curves for each assay at the specification of samples taken ≥20 post symptom onset The green shaded areas represent a target sensitivity and specificity of at least 98%. Dashed lines show the 98% sensitivity and specificity thresholds used as the standard. Assay threshold values associated with ten exemplar points on the ROC curve are shown in each panel. Data are presented for 976 known negative samples and 536 known positive samples run on each assay. ROC=receiver operating characteristic. Abbott=SARS-CoV-2 IgG assay (Abbott, Chicago, IL, USA). DiaSorin=LIAISON SARS-CoV-2 S1/S2 IgG assay (DiaSorin, Saluggia, Italy). Oxford immunoassay=a novel 384-well format ELISA (University of Oxford, Oxford, UK). Roche=Elecsys Anti-SARS-CoV-2 assay (Roche, Basel, Switzerland). SARS-CoV-2=severe acute respiratory syndrome coronavirus 2. Siemens=SARS-CoV-2 Total assay (Siemens, Munich, Germany).

Using the pre-defined assay thresholds for calling test results positive or negative, only the Siemens assay and the Oxford immunoassay met the specificity and sensitivity target of at least 98%, achieving this threshold at all three timepoints assessed (≥14, ≥20, and ≥30 days post symptom onset; appendix pp 28, 29; figure 1; table). The Roche assay also met the specificity and sensitivity target of at least 98% on samples taken at least 30 days post symptom onset (appendix p 29; table). ROC curve analysis showed that the Roche assay could also potentially meet the sensitivity and specificity targets on samples taken at least 14 and at least 20 days post symptom onset through threshold adjustment—eg, adjusting the threshold to at least 0·128 as opposed to the current threshold of more than 1·0 would result in a sensitivity and specificity of at least 98% (with a lower bound of the 95% CI of ≥96%) at 20 days or more post symptom onset (figure 3; appendix p 30 for samples taken ≥14 days post symptom onset; table). On samples taken at least 30 days post symptom onset (but not ≥14 or ≥20 days post symptom onset), the Abbott and DiaSorin assay thresholds could be optimised to achieve sensitivity and specificity of at least 98% (with a lower bound of ≥96%; appendix p 31; table). Overall, on samples taken at least 30 days post symptom onset, optimisation of assay thresholds to ensure a specificity of at least 98% (with a lower bound of ≥96%) would result in sensitivities of at least 98% (with a lower bound of ≥96%) for all assays assessed (appendix pp 31, 32; table).

The NHSBT standards (10061-63) correlated well with the NIBSC reagents using the Oxford immunoassay (appendix p 10). Replicate testing of serial dilutions of the NHSBT standards showed good reproducibility, albeit with some interesting differences observed—eg, for nucleocapsid protein assays (Abbott and Roche), readings for the NHSBT medium control (10061) dilution series were lower than those of the NHSBT low control (10063), suggesting that antibodies targeting nucleocapsid and spike proteins might be present at different titres in certain samples (appendix p 33). For the Roche and Siemens assays the entire high control dilution series generated values above the assay thresholds for calling positives. For the Abbott assay, all of the medium titre dilution series generated values below the threshold for distinguishing positives.

**Figure 4:**
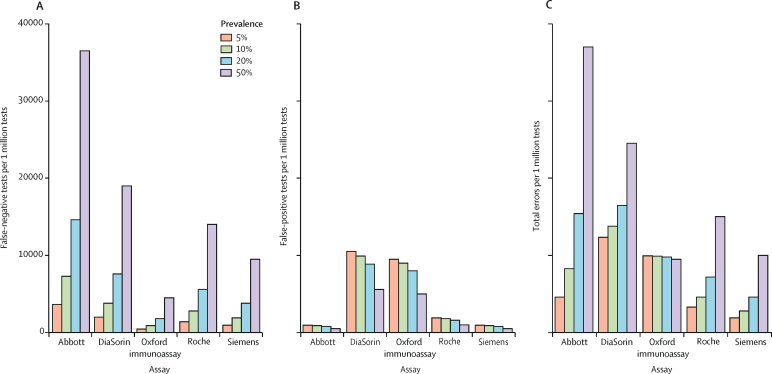
False negatives, false positives, and total errors per 1 million tests False negatives (A), false positives (B) and total errors (C) per 1 million tests, using the unadjusted thresholds (manufacturers, and Oxford immunoassay), and sensitivity and specificity for all assays for samples tested after at least 20 days post symptom onset, at population SARS-CoV-2 seroprevalence of 5%, 10%, 20%, and 50%. Abbott=SARS-CoV-2 IgG assay (Abbott, Chicago, IL, USA). DiaSorin=LIAISON SARS-CoV-2 S1/S2 IgG assay (DiaSorin, Saluggia, Italy). Oxford immunoassay=a novel 384-well format ELISA (University of Oxford, Oxford, UK). Roche=Elecsys Anti-SARS-CoV-2 assay (Roche, Basel, Switzerland). SARS-CoV-2=severe acute respiratory syndrome coronavirus 2. SARS-CoV-2=severe acute respiratory syndrome coronavirus 2. Siemens=SARS-CoV-2 Total assay (Siemens, Munich, Germany).

Although sensitivity and specificity are useful, positive and negative predictive values are more directly relevant to public health and clinical application. At 10% prevalence, the high specificity platforms (Abbott, Roche, and Siemens assays) had lower numbers of false positive tests per million tests compared with other assays (DiaSorin and Oxford immunoassay; figure 4; appendix p 21). However, the number of false negative tests per million was higher with the Abbott and DiaSorin assays and lowest with the Oxford immunoassay (figure 4; appendix p 21). Overall, the Siemens assay had the fewest total errors per million tests for prevalences of 20% or lower (figure 4; appendix p 21).

Antibody responses increased over the first 3–4 weeks after symptom onset, with the maximum percentage of positive tests reached by day 27 across all assays (appendix p 34). Antibody responses were sustained up to 73 days post symptom onset and up to 82 days post positive RT-PCR result (appendix p 35); however, any declines in titres could not be specifically ruled out given that, by this stage, most quantitative results were at near-maximum values for each assay.

For 158 (29%) of 536 individuals in the positive cohort for whom disease severity data were available, we found no evidence of a difference in immunoassay sensitivity by disease severity (asymptomatic n=13 [8%], mild n=122 [77%], severe n=16 [10%], critical or death n=7 [4%]) for any of the assays assessed (appendix p 36); however numbers were small in the non-mild groups.

We also undertook an analysis of discordance between assays (appendix pp 22, 23). Most of the discordances were observed in samples taken before 20 days post symptom onset for SARS-CoV-2-positive patients confirmed by RT-PCR (37 [30%] of 124 samples taken <20 days post symptom onset *vs* 46 [9%] 538 samples taken ≥20 days post symptom onset; 27 [3%] of 976 pre-pandemic samples; p<0·0001). Three (<1%) of the samples classified as known positive (from two mild and one severe case) tested antibody-negative across all assays.

## Discussion

On a large panel of blood samples, we showed that the Siemens assay and the Oxford immunoassay both achieved sensitivity and specificity of at least 98% (with a lower bound of the 95% CI ≥96%) on samples taken at least 20 days post symptom onset without optimisation. However, all assays could potentially achieve these specifications through threshold adjustment, or by assessing samples collected at least 30 days post symptom onset, consistent with the time-dependent nature of antibody responses. Additionally, we highlighted the potentially scalable capacity of the Oxford immunoassay, which could enable centralised testing for large-scale projects (eg, national seroprevalence studies). This finding suggests that global serology testing needs can be met using different assays, mitigating against the risk of shortages, and allowing immediate deployment in laboratories with different analysers already installed for other testing purposes. The optimal deployment of assays should reflect use case—eg, an optimised assay measuring antibodies to nucleocapsid protein might be most relevant for disease surveillance in the context of a widespread rollout of vaccines, which are mostly spike-protein based.

Although sensitivity and specificity are important, estimating the negative and positive predictive values of each assay are perhaps more useful in deciding how tests can best be used and interpreted. Most published seroprevalence studies of non-health-care workers have estimated antibody prevalences of less than 15%.^22^, ^23^ From our estimates, all assays would have an error rate of less than 1% at a seroprevalence of 10%, except for the DiaSorin assay. Nevertheless, this error rate would still translate into several thousand errors per million tests done for any of the assays investigated, which could have important implications at an individual level.

In our study, antibody titres within individuals were largely sustained beyond 2 months of symptom onset for all assays investigated, suggesting that serosurveillance with these assays is likely to be accurate within months of exposure at least. We did not observe any significant difference in assay sensitivity by disease severity; notably, however, the numbers in our non-mild disease category, including asymptomatic cases, were small, and we were limited by the bounds determined by the dynamic range of the assays and saturation. Inconsistencies have been observed in the association between antibody titres and disease severity in other studies, which might relate to assay target.^20^, ^24^, ^25^ Further studies definitively assessing the longer-term trajectory of antibodies in larger longitudinal cohorts of individuals with different disease severities and taking the specific assay type into account would be of benefit.

Although all these assays can effectively detect SARS-CoV-2 antibodies, the durability and nature of immunity conferred by these antibodies remains unclear. Few data are available on immunity to seasonal human coronaviruses (alphacoronaviruses NL63 and 229E, and betacoronaviruses HKU1 and OC43). In two small, controlled, human infection models, re-challenge at 8–12 months with 229E (nine individuals)^26^ and 229E-related strains (six)^27^ showed protection against symptoms, but virus shedding was detected in six (67%) of nine individuals.^26^ In an observational study of NL63 in Kenya, reinfections over a 6-month period occurred in 46 (28%) of 163 individuals, with reinfections within 80 days largely associated (>80% cases) with lower viral titres and fewer symptoms compared with those associated with reinfections after more than 80 days.^28^ Data on Middle East respiratory syndrome (MERS) and severe acute respiratory syndrome coronavirus suggest that antibody titres wane over periods of approximately 2 years, with more severe infection resulting in more measurable and durable antibody responses in MERS infection.^29^, ^30^ Animal data (re-challenge in four rhesus macaques) suggest protection from re-infection with SARS-CoV-2 extends to at least 28 days post infection.^31^ The relationship between SARS-CoV-2 antibodies and neutralising activity remains an important research question. Because the spike protein is responsible for enabling cell entry, the spike protein appears to be the main antigen responsible for eliciting neutralising antibodies. In SARS-CoV-2, a correlation between anti-spike antibody titres when trimeric versions of spike are used as the antigen and neutralising capacity appears to be emerging.^24^, ^32^ If SARS-CoV-2 antibody titres are shown to clearly correlate directly or indirectly with immunity to infection or attenuation of disease severity, then more accurately evaluating titres by optimising the dynamic range of assays might become relevant.

Notably, no clear gold standard exists against which to assess these antibody tests. RT-PCR positivity is a proxy for the expected presence of antibody, but negative antibody tests in RT-PCR-positive individuals could either reflect antibody test performance, or alternatively be explained by a failure to mount a measurable systemic IgM or IgG antibody response (eg, in cases of immunocompromised individuals), effective infection clearance through other immune mechanisms (eg, T cells), an issue with the sample (eg, a major interferent), or through a false-positive RT-PCR test in individuals who have not had SARS-CoV-2 infection. The three samples that were negative by all assays in our study could plausibly have had a biological absence of antibodies.

Our study was limited by sample volumes, especially given the constraints imposed by dead volume requirements for liquid handling, and we were unable to do repeat analyses. Subgroup analysis by timing of collection or disease severity was constrained by small numbers and larger studies would be of value. Neutralisation and pseudo-neutralisation assays provide an in-vitro approach to investigating antibodies to help address the question as to whether antibody titres correlate with functionality; this work is ongoing. Longitudinal prospective clinical studies are also needed to investigate whether repeat infections can arise in antibody-positive individuals. Our sample sets might under-represent some ethnic groups, did not include children, and captured insufficient clinical meta-data to characterise features (eg, being immunocompromised), which might explain the presence of false-negative antibody results in individuals with RT-PCR-confirmed infection. Additional performance assessments in these groups would be warranted. Although assessments of precision and reproducibility have to some extent been carried out by the immunoassay manufacturers, a complete validation of the individual assays was beyond the scope of our study. We did not investigate our large negative sample set for the presence of antibodies against seasonal coronaviruses, the presence of which might have accounted for some of the false-positive results. However, previous manufacturers' assessments for the Abbott and Roche immunoassays, and our assessment as part of this study for the Oxford immunoassay on small panels of sera taken from patients with respiratory virus infections including seasonal coronavirus infections, suggest good analytical specificity.

In conclusion, we characterised the performance of four commercial antibody platforms and a new ELISA from an academic research facility, all of which achieved the desired specificity, and with opportunities to optimise sensitivity for the Abbott, DiaSorin, and Roche assays through changes to thresholds and sample timing, although any post-hoc changes to thresholds would require repeat verification of sensitivity and specificity. Assay selection should carefully reflect use case. This study represents a benchmark for future assessments of serological platforms. Such assays will be an important part of the clinical and research landscape in guiding public health policy, with effects to be delivered at the individual level and population level.

**This online publication has been corrected. The corrected version first appeared at thelancet.com/infection on November 25, 2020**

## Data sharing

Available metadata for each sample in the study, including raw immunoassay results and interpretation, are presented as online datasets.
