# Supplementary material for: Performance characteristics of five immunoassays for SARS-CoV-2: a head-to-head benchmark comparison
Source: Lancet Infect Dis. 2020 Dec;20(12):1390–400. doi: 10.1016/S1473-3099(20)30634-4 (PMC7511171; doi:10.1016/S1473-3099(20)30634-4)
Supplement: Supplementary appendix [file mmc1.pdf]

THE LANCET
Infectious Diseases

## Supplementary appendix

This appendix formed part of the original submission and has been peer reviewed. We post it as supplied by the authors.

This online publication has been corrected. The corrected version first appeared at thelancet.com on Nov 25, 2020.

Supplement to: The National SARS-CoV-2 Serology Assay Evaluation Group. Performance characteristics of five immunoassays for SARS-CoV-2: a head-to-head benchmark comparison. *Lancet Infect Dis* 2020; published online Sept 23. https://doi.org/10.1016/S1473-3099(20)30634-4.

# Supplementary material

## The National SARS-CoV-2 Serology Assay Evaluation Group (complete, alphabetical listing)

| Surname | First name/subsequent initials | Affiliation |
|---|---|---|
| Ainsworth | Mark | Oxford University Hospitals NHS Foundation Trust, Oxford, UK |
| Andersson | Monique | Oxford University Hospitals NHS Foundation Trust, Oxford, UK |
| Auckland | Kathryn | Wellcome Centre for Human Genetics, Nuffield Department of Medicine (NDM), University of Oxford, Oxford, UK |
| Baillie | J. Kenneth | University of Edinburgh, Edinburgh, UK |
| Barnes | Eleanor | Nuffield Department of Medicine, University of Oxford, Oxford, UK |
| Beer | Sally | Oxford University Hospitals NHS Foundation Trust, Oxford, UK |
| Beveridge | Amy | Oxford Vaccine Group, Department of Paediatrics, University of Oxford, Oxford, UK |
| Bibi | Sagida | Oxford Vaccine Group, Department of Paediatrics, University of Oxford, Oxford, UK |
| Blackwell | Luke | Oxford Vaccine Group, Department of Paediatrics, University of Oxford, Oxford, UK |
| Borak | Martyna | University of Oxford, Oxford, UK |
| Bown | Abbie | Public Health England (PHE) Porton Down, Salisbury, UK |
| Brooks | Tim | Public Health England (PHE) Porton Down, Salisbury, UK |
| Burgess-Brown | Nicola A. | Structural Genomics Consortium, Nuffield Department of Medicine, University of Oxford, Oxford, UK |
| Camara | Susana | Oxford Vaccine Group, Department of Paediatrics, University of Oxford, Oxford, UK |
| Catton | Matthew | Public Health England (PHE) Porton Down, Salisbury, UK |
| Chau | Kevin K. | Nuffield Department of Medicine, University of Oxford, Oxford, UK |
| Christott | Thomas | The Structural Genomics Consortium, Nuffield Department of Medicine (NDM), University of Oxford and the Target Discovery Institute, Nuffield Department of Medicine, University of Oxford, Oxford, UK |
| Clutterbuck | Elizabeth | Department of Paediatrics, University of Oxford, UK |
| Coker | Jesse | The Structural Genomics Consortium, Nuffield Department of Medicine (NDM), University of Oxford, Oxford, UK |
| Cornall | Richard J. | Nuffield Department of Medicine, University of Oxford, Oxford, UK |
| Cox | Stuart | Oxford University Hospitals NHS Foundation Trust, Oxford, Oxford, UK |
| Crawford-Jones | David | Oxford University Hospitals NHS Foundation Trust, Oxford, Oxford, UK |
| Crook | Derrick W. | Nuffield Department of Medicine, University of Oxford, Oxford, UK |
| D’Arcangelo | Silvia | Public Health England (PHE) Porton Down, Salisbury, UK |
| Dejnirattsai | Wanwisa | Wellcome Centre for Human Genetics, Nuffield Department of Medicine (NDM), University of Oxford, Oxford, UK |
| Dequaire | Julie M.M. | University of Oxford, Oxford, UK |
| Dimitriadis | Stavros | University of Oxford, Oxford, UK |
| Dingle | Kate E. | Nuffield Department of Medicine, |

| | | |
|---|---|---|
| | | University of Oxford, Oxford, UK |
| Doherty | George | Nuffield Department of Medicine, University of Oxford, Oxford, UK |
| Dold | Christina | Oxford Vaccine Group, Department of Paediatrics, University of Oxford and the NIHR Oxford Biomedical Research Centre, University of Oxford, Oxford, UK |
| Dong | Tao | MRC Human Immunology Unit, MRC Weatherall Institute of Molecular Medicine, University of Oxford, Oxford, UK and the Chinese Academy of Medical Science(CAMS) Oxford Institute (COI), University of Oxford, Oxford, UK |
| Dunachie | Susanna J. | Centre for Tropical Medicine & Global Health, University of Oxford, Oxford, UK |
| Ebner | Daniel | The Target Discovery Institute, Nuffield Department of Medicine, University of Oxford, Oxford, UK |
| Emmenegger | Marc | Institute of Neuropathology, University of Zuerich, Zuerich, Switzerland |
| Espinosa | Alexis | Oxford University Hospitals NHS Foundation Trust, Oxford, Oxford, UK |
| Eyre | David W. | Big Data Institute, Nuffield Department of Population Health, University of Oxford, Oxford, UK |
| Fairhead | Rory | University of Oxford, Oxford, UK |
| Fassih | Shayan | University of Oxford, Oxford, UK |
| Feehily | Conor | Teagasc, Moorepark Food Research Centre, Fermoy, Co. Cork, Ireland and APC Microbiome Ireland, University College Cork, Cork, Ireland |
| Felle | Sally | Oxford Vaccine Group, Department of Paediatrics, University of Oxford, Oxford, UK |
| Fernandez-Cid | Alejandra | The Structural Genomics Consortium, Nuffield Department of Medicine (NDM), University of Oxford, Oxford, UK |
| Fernandez Mendoza | Maria | Oxford University Hospitals NHS Foundation Trust, Oxford, Oxford, UK |
| Foord | Thomas H. | University of Oxford, UK |
| Fordwoh | Thomas | Oxford University Hospitals NHS Foundation Trust, Oxford, Oxford, UK |
| Fox McKee | Deborah | Public Health England (PHE) Porton Down, Salisbury, UK |
| Frater | John | Nuffield Department of Medicine, University of Oxford, Oxford, UK and NIHR Oxford Biomedical Research Centre |
| Gallardo Sanchez | Veronica | Oxford University Hospitals NHS Foundation Trust, Oxford, Oxford, UK |
| Gent | Nick | Public Health England (PHE) Porton Down, Salisbury, UK |
| Georgiou | Dominique | Oxford University Hospitals NHS Foundation Trust, Oxford, Oxford, UK |
| Groves | Christopher J. | Nuffield Department of Medicine, University of Oxford, Oxford, UK |
| Hallis | Bassam | Public Health England (PHE) Porton Down, Salisbury, UK |
| Hammond | Peter M. | Public Health England (PHE) Porton Down, Salisbury, UK |
| Hatch | Stephanie B. | The Target Discovery Institute, Nuffield Department of Medicine, University of Oxford, Oxford, UK |
| Harvala | Heli J. | NHS Blood and Transplant, UK |

| | | |
|---|---|---|
| Hill | Jennifer | Oxford Vaccine Group, Department of Paediatrics, University of Oxford, Oxford, UK |
| Hoosdally | Sarah J. | Nuffield Department of Medicine, University of Oxford, Oxford, UK |
| Horsington | Bryn | Oxford University Hospitals NHS Foundation Trust, Oxford, Oxford, UK |
| Howarth | Alison | The Structural Genomics Consortium, Nuffield Department of Medicine (NDM), University of Oxford, Oxford, UK |
| James | Tim | Oxford University Hospitals NHS Foundation Trust, Oxford, UK |
| Jeffery | Katie | Oxford University Hospitals NHS Foundation Trust, Oxford, Oxford, UK |
| Jones | Elizabeth | Department of Paediatrics, University of Oxford, Oxford, UK |
| Justice | Anita | Oxford University Hospitals NHS Foundation Trust, Oxford, Oxford, UK |
| Karpe | Fredrik | Oxford Centre for Diabetes, Endocrinology and Metabolism, Radcliffe Department of Medicine, University of Oxford, Oxford, UK |
| Kavanagh | James | Nuffield Department of Medicine, University of Oxford, Oxford, UK |
| Kim | David S. | University of Oxford, UK |
| Kirton | Richard | Oxford University Hospitals NHS Foundation Trust, Oxford, Oxford, UK |
| Klenerman | Paul | The Peter Medawar Building for Pathogen Research, University of Oxford, Oxford, UK |
| Knight | Julian C. | Wellcome Centre for Human Genetics, Nuffield Department of Medicine (NDM), University of Oxford, Oxford, UK |
| Koukouflis | Leonidas | The Structural Genomics Consortium, Nuffield Department of Medicine (NDM), University of Oxford, Oxford, UK |
| Kwok | Andrew | Wellcome Centre for Human Genetics, Nuffield Department of Medicine, University of Oxford, Oxford, UK |
| Leuschner | Ullrich | University of Oxford, Oxford, UK |
| Levin | Robert | Worthing Hospital, Worthing, UK |
| Linder | Aline | Department of Paediatrics, University of Oxford, Oxford, UK |
| Lockett | Teresa | Oxford University Hospitals NHS Foundation Trust, Oxford, Oxford, UK |
| Lumley | Sheila F. | Oxford University Hospitals NHS Foundation Trust and Nuffield Department of Medicine, University of Oxford, Oxford, UK |
| Marinou | Spyridoula | Oxford Vaccine Group, Department of Paediatrics, University of Oxford, Oxford, UK |
| Marsden | Brian D. | The Structural Genomics Consortium, Nuffield Department of Medicine (NDM), University of Oxford and the Kennedy Institute, Nuffield Department of Orthopaedics, Rheumatology and Musculoskeletal Sciences (NDORMS), University of Oxford, Oxford, UK |
| Martinez | Jose | Oxford University Hospitals NHS Foundation Trust, Oxford, Oxford, UK |
| Martins Ferreira | Lucas | The Structural Genomics Consortium, Nuffield Department of Medicine |

| | | |
|---|---|---|
| | | (NDM), University of Oxford, Oxford, UK |
| Mason | Lara | Public Health England (PHE) Porton Down, Salisbury, UK |
| Matthews | Philippa C. | Nuffield Department of Medicine, University of Oxford, Oxford, UK |
| Mentzer | Alexander J. | Wellcome Centre for Human Genetics, Nuffield Department of Medicine, University of Oxford, Oxford, UK |
| Mobbs | Alexander | Oxford University Hospitals NHS Foundation Trust, Oxford, Oxford, UK |
| Mongkolsapaya | Juthathip | Wellcome Centre for Human Genetics, Nuffield Department of Medicine, University of Oxford, Oxford, UK and the Dengue Hemorrhagic Fever Research Unit, Office for Research and Development, Siriraj Hospital, Faculty of Medicine, Mahidol Univeristy, Bangkok, Thailand |
| Morrow | Jordan | Oxford University Hospitals NHS Foundation Trust, Oxford, Oxford, UK |
| Mukhopadhyay | Shubhashish M. M. | Structural Genomics Consortium, Nuffield Department of Medicine, University of Oxford, Oxford, UK |
| Neville | Matthew J. | Oxford Centre for Diabetes, Endocrinology and Metabolism, Radcliffe Department of Medicine, University of Oxford, Oxford, UK |
| Oakley | Sarah | Oxford University Hospitals NHS Foundation Trust, Oxford, Oxford, UK |
| Oliveira | Marta | Nuffield Department of Surgical Sciences, University of Oxford, Oxford, UK |
| Otter | Ashley | Public Health England (PHE) Porton Down, Salisbury, UK |
| Paddon | Kevin | Oxford University Hospitals NHS Foundation Trust, Oxford, Oxford, UK |
| Pascoe | Jordan | Public Health England (PHE) Porton Down, Salisbury, UK |
| Peng | Yanchun | MRC Human Immunology Unit, MRC Weatherall Institute of Molecular Medicine, University of Oxford, Oxford, UK and Chinese Academy of Medical Science (CAMS) Oxford Institute (COI), University of Oxford, Oxford, UK |
| Perez | Elena | Oxford University Hospitals NHS Foundation Trust, Oxford, Oxford, UK |
| Perumal | Prem K. | Public Health England (PHE) Porton Down, Salisbury, UK |
| Peto | Timothy E.A. | Nuffield Department of Medicine, University of Oxford, Oxford, UK |
| Pickford | Hayleah | Nuffield Department of Medicine, University of Oxford, Oxford, UK |
| Ploeg | Rutger J. | Nuffield Department of Surgical Sciences, University of Oxford, Oxford, UK and NHS Blood & Transplant, UK and Leiden University, The Netherlands |
| Pollard | Andrew J. | Oxford Vaccine Group, Department of Paediatrics University of Oxford and the NIHR Oxford Biomedical Research Centre, Oxford, UK |
| Richardson | Anastasia | Public Health England (PHE) Porton Down, Salisbury, UK |
| Ritter | Thomas G. | University of Oxford, Oxford, UK |
| Roberts | David J. | NHS Blood and Transplant, UK |

| | | |
|---|---|---|
| Rodger | Gillian | Nuffield Department of Medicine, University of Oxford, Oxford, UK |
| Rollier | Christine S. | Oxford Vaccine Group, Department of Paediatrics, University of Oxford, Oxford, UK |
| Rowe | Cathy | Public Health England (PHE) Porton Down, Salisbury, UK |
| Rudkin | Justine K. | Big Data Institute, Nuffield Department of Population Health, University of Oxford, Oxford, Oxford, UK |
| Screaton | Gavin | Nuffield Department of Medicine, University of Oxford, Oxford, UK |
| Semple | Malcolm G. | University of Liverpool, Liverpool, UK |
| Sienkiewicz | Alex | Public Health England (PHE) Porton Down, Salisbury, UK |
| Silva-Reyes | Laura | Oxford Vaccine Group, Department of Paediatrics, University of Oxford, Oxford, UK |
| Skelly | Donal T. | Nuffield Department of Clinical Neurosciences, University of Oxford, Oxford, UK |
| Sobrino Diaz | Alberto | Oxford University Hospitals NHS Foundation Trust, Oxford, Oxford, UK |
| Stafford | Lizzie | Oxford University Hospitals NHS Foundation Trust, Oxford, UK and NIHR Oxford Biomedical Research Centre, Oxford, Oxford, UK |
| Stockdale | Lisa | Oxford Vaccine Group, Department of Paediatrics, University of Oxford, Oxford, UK |
| Stoesser | Nicole | Nuffield Department of Medicine, University of Oxford, Oxford, UK |
| Street | Teresa | Nuffield Department of Medicine, University of Oxford, Oxford, UK |
| Stuart | David I. | The Division of Structural Biology (STRUBI), Nuffield Department of Medicine, University of Oxford, Oxford, UK and and Diamond Light Source Ltd, Harwell Science & Innovation Campus, Didcot, UK |
| Sweed | Angela | Public Health England (PHE) Porton Down, Salisbury, UK |
| Taylor | Adan | University of Oxford, Oxford, UK |
| Thraves | Hannah | Oxford University Hospitals NHS Foundation Trust, Oxford, Oxford, UK |
| Tsang | Hoi P. | NHS Blood and Transplant, UK |
| Verheul | Marije K. | Oxford Vaccine Group, Department of Paediatrics, University of Oxford, UK and NIHR Oxford Biomedical Research Centre, Oxford, UK |
| Vipond | Richard | Public Health England (PHE) Porton Down, Salisbury, UK |
| Walker | Timothy M. | Nuffield Department of Medicine, University of Oxford, Oxford, UK and Oxford University Clinical Research Unit, Ho Chi Minh City, Vietnam |
| Wareing | Susan | Oxford University Hospitals NHS Foundation Trust, Oxford, UK |
| Warren | Yolanda | Oxford University Hospitals NHS Foundation Trust, Oxford, UK |
| Wells | Charlie | Oxford University Hospitals NHS Foundation Trust, Oxford, UK |
| Wilson | Clare | Public Health England (PHE) Porton Down, Salisbury, UK |
| Withycombe | Kate | Public Health England (PHE) Porton Down, Salisbury, UK |
| Young | Rebecca K. | University of Oxford, Oxford, UK |

***Complete Author contributions (as per CRediT; Contributor Roles Taxonomy)***
**Conceptualization** - A Bown, T Brooks, DW Crook, DW Eyre, B Hallis, P Hammond, T James, PC Matthews, TEA Peto, C Rowe, A Sienkiewicz, N Stoesser, R Vipond
**Methodology** - A Bown, T Brooks, DW Crook, DW Eyre, B Hallis, P Hammond, T James, PC Matthews, TEA Peto, C Rowe, A Sienkiewicz, N Stoesser, R Vipond
**Software** - T Christott, DW Eyre, L Koukouflis, BD Marsden, L Martins Ferreira, K Paddon
**Validation** - A Bown, T Brooks, S Cox, DW Crook, W Dejnirattsai, D Ebner, M Emmenegger, DW Eyre, B Hallis, P Hammond, SB Hatch, A Howarth, T James, PC Matthews, J Mongkolsapaya, TEA Peto, C Rowe, A Sienkiewicz, N Stoesser, R Vipond
**Formal analysis** - DW Eyre, N Gent, PC Matthews, TEA Peto, N Stoesser
**Investigation** - K Auckland, A Beveridge, S Bibi, L Blackwell, A Bown, T Brooks, S Camara, M Catton, KK Chau, T Christott, E Clutterbuck, D Crawford-Jones, S Cox, C Dold, S D'Arcangelo, W Dejnirattsai, J Dequaire, KE Dingle, G Doherty, D Ebner, A Espinosa, DW Eyre, C Feehily, S Felle, D Fox McKee, B Hallis, P Hammond, J Hill, SJ Hoosdally, A Howarth, J Kavanagh, R Kirton, A Linder, SF Lumley, S Marinou, L Mason, BD Marsden, J Martinez, L Martins Ferreira, PC Matthews, AJ Mentzer, A Mobbs, J Mongkolsapaya, S Oakley, M Oliveira, A Otter, J Pascoe, PK Perumal, H Pickford, A Richardson, G Rodger, CS Rollier, C Rowe, JK Rudkin, A Sienkiewicz, L Silva-Reyes, M Spyridoula, L Stafford, T Street, N Stoesser, A Sweed, MK Verheul, R Vipond, C Wilson, K Withycombe
**Resources** - M Ainsworth, M Andersson, JK Baillie, E Barnes, S Beer, M Borak, A Bown, T Brooks, NA Burgess-Brown, J Coker, R Cornall, DW Crook, S Dimitriadis, T Dong, SJ Dunachie, D Ebner, DW Eyre, R Fairhead, S Fassih, A Fernandez-Cid, M Fernandez Mendoza, TH Foord, T Fordwoh, J Frater, V Gallardo Sanchez, D Georgiou, CJ Groves, B Hallis, P Hammond, HJ Harvala, B Horsington, T James, E Jones, K Jeffery, A Justice, F Karpe, DS Kim, P Klenerman, JC Knight, U Leuschner, T Lockett, J Martinez, PC Matthews, AJ Mentzer, J Mongkolsapaya, SMM Mukhopadhyay, MJ Neville, S Oakley, M Oliveira, Elena Perez, RJ Ploeg, AJ Pollard, TG Ritter, DJ Roberts, C Rowe, CS Rollier, C Rowe, G Screaton, MG Semple, A Sienkiewicz, DT Skelly, A Sobrino Diaz, L Stockdale, DI Stuart, N Stoesser, A Taylor, H Thraves, HP Tsang, S Wareing, R Vipond, TM Walker, Y Warren, C Wells, R Young
**Data Curation** - A Bown, T Christott, D Ebner, DW Eyre, SB Hatch, A Howarth, L Koukouflis, BD Marsden, L Martins Ferreira, PC Matthews, AJ Mentzer, MJ Neville, C Rowe, N Stoesser
**Writing - Original draft** - DW Eyre, PC Matthews, N Stoesser
**Writing - Review & Editing** - All authors
**Visualization** - DW Eyre, PC Matthews, TEA Peto, N Stoesser
**Supervision** - A Bown, T Brooks, R Cornall, DW Crook, D Ebner, DW Eyre, B Hallis, P Hammond, SB Hatch, A Howarth, T James, BD Marsden, PC Matthews, TEA Peto, C Rowe, A Sienkiewicz, N Stoesser, DI Stuart, R Vipond
**Project administration -** A Bown, T Brooks, DW Crook, D Ebner, DW Eyre, B Hallis, P Hammond, SB Hatch, SJ Hoosdally, A Howarth, T James, BD Marsden, PC Matthews, TEA Peto, A Sienkiewicz, N Stoesser, G Screaton, DI Stuart, R Vipond
**Funding acquisition** - R Cornall, DW Crook, G Screaton, A Sienkiewicz, DI Stuart

**All authors - Declaration of Interests**
Richard Cornall is a founder of MIROBIO and reports reports personal fees and other from MIROBio, outside of the submitted work.
David W. Eyre has received lecture fees from Gilead, outside of the submitted work.
Malcolm G. Semple reports grants from the Department of Health and Social Care (DHSC), National Institute of Health Research UK, grants from Medical Research Council UK, grants from Health Protection Research Unit in Emerging & Zoonotic Infections, University of Liverpool, during the conduct of the study; other from Integrum Scientific LLC, Greensboro, NC, USA, outside of the submitted work.

**Additional acknowledgements**
We are grateful to all the participants and patients who have contributed samples, and all of the biomedical scientists who have processed samples for this study. This work uses data provided by patients and collected by the NHS as part of their care and support #DataSavesLives. We are extremely grateful to the 2,648 frontline NHS clinical and research staff and volunteer medical students, who collected this data in challenging circumstances; and the generosity of the participants and their families for their individual contributions in these difficult times.

**Additional funding statements**
This work is also supported by grants from: the National Institute for Health Research [award CO-CIN-01], the Medical Research Council [grant MC_PC_19059] and by the National Institute for Health Research Health

Protection Research Unit (NIHR HPRU) in Emerging and Zoonotic Infections at University of Liverpool in partnership with Public Health England (PHE), in collaboration with Liverpool School of Tropical Medicine and the University of Oxford [NIHR award 200907], Wellcome Trust and Department for International Development [215091/Z/18/Z], and the Bill and Melinda Gates Foundation [OPP1209135], and Liverpool Experimental Cancer Medicine Centre for providing infrastructure support for this research (Grant Reference: C18616/A25153). The views expressed are those of the authors and not necessarily those of the DHSC, DID, NIHR, MRC, Wellcome Trust or PHE.

Eleanor Barnes is supported by the Oxford NIHR Biomedical Research Centre and is an NIHR Senior Investigator.
Kevin K. Chau is a Medical Research Foundation National PhD Training Programme student (MRF-145-0004-TPG-AVISO).
Christina Dold is funded by Innovate UK.
Tao Dong is funded by the Medical Research Council and the Chinese Academy of Medical Sciences (CAMS) Innovation Fund for Medical Sciences (CIFMS), China (grant number: 2018-I2M-2-002).
Paul Klenerman is a Wellcome Trust Investigator (WT109965MA) and holds an NIHR Senior Fellowship.
Julian C. Knight is a Wellcome Trust Investigator (204969/Z/16/Z) and receives support from the NIHR Oxford Biomedical Research Centre.
Jesse Coker is supported by the Kennedy Trust for Rheumatology Research and SGC, a registered charity (number 1097737) that receives funds from AbbVie, Bayer Pharma AG, Boehringer Ingelheim, Canada Foundation for Innovation, Eshelman Institute for Innovation, Genome Canada through Ontario Genomics Institute [OGI-055], Innovative Medicines Initiative (EU/EFPIA) [ULTRA-DD grant no. 115766], Janssen, Merck KGaA, Darmstadt, Germany, MSD, Novartis Pharma AG, Pfizer, São Paulo Research Foundation-FAPESP, Takeda, and Wellcome.
Nicola A. Burgess-Brown, Alejandra Fernandez-Cid, Lucas Martins Ferreira, Shubhashish Mukhopadhyay and Thomas Christott are supported by the SGC, a registered charity (number 1097737) that receives funds from AbbVie, Bayer Pharma AG, Boehringer Ingelheim, Canada Foundation for Innovation, Eshelman Institute for Innovation, Genome Canada through Ontario Genomics Institute [OGI-055], Innovative Medicines Initiative (EU/EFPIA) [ULTRA-DD grant no. 115766], Janssen, Merck KGaA, Darmstadt, Germany, MSD, Novartis Pharma AG, Pfizer, São Paulo Research Foundation-FAPESP, Takeda, and Wellcome.
Yanchun Peng is funded by the Medical Research Council and the Chinese Academy of Medical Sciences (CAMS) Innovation Fund for Medical Sciences (CIFMS), China (grant number: 2018-I2M-2-002).
Rutger Ploeg is supported by funding from the NIHR Oxford Biomedical Research Centre, the Medical Research Council, NHS Blood and Transplant, the NIHR.
Alex J. Mentzer is supported by the NIHR and the NIHR Oxford Biomedical Research Centre.
Christine S. Rollier is supported by the NIHR Oxford Biomedical Research Centre.
Justine K. Rudkin is supported by a Sir Henry Dale Fellowship, jointly funded by the Wellcome Trust and the Royal Society (Grant 101237/Z/13/B).
Donal T. Skelly is supported by the NIHR. The views expressed in this article are those of the author and not necessarily those of the NHS, the NIHR, or the Department of Health.
Timothy M. Walker is a Wellcome Trust Clinical Career Development Fellow (214560/Z/18/Z).
Yolanda Warren is funded by the University of Oxford and the NIHR Clinical Research Network: Thames Valley and South Midlands.

**Supplementary Methods**
**A. Systematic review**
We considered any article published in English, German, French, Spanish or Italian. Screening was performed by a single reviewer, using a screening template to exclude studies if they were not focused on determining SARS-CoV-2 serology, did not evaluate sensitivity/specificity for (an) assay(s), or did not evaluate assays included in our head-to-head; replicate references across databases were de-duplicated. Data extraction for relevant studies was completed by two extractors using a template table (in line with PICOS [Participants, Interventions, Comparisons, Outcomes, Study design]) which included publication details, a description of the cohort samples investigated, the sample sizes used to generate metrics, sensitivity and specificity for the relevant assay being evaluated, and additional freetext notes (see Supplementary Methods/Supplementary dataset SD1/PRISMA checklist).

The expanded search terms in PubMed were as follows:
(("severe acute respiratory syndrome coronavirus 2"[Supplementary Concept] OR "severe acute respiratory syndrome coronavirus 2"[All Fields]) OR "sars cov 2"[All Fields]) AND ((((((((("elisa s"[All Fields] OR

"elisas"[All Fields]) OR "enzyme-linked immunosorbent assay"[MeSH Terms]) OR (("enzyme linked"[All Fields] AND "immunosorbent"[All Fields]) AND "assay"[All Fields])) OR "enzyme linked immunosorbent assay"[All Fields]) OR "elisa"[All Fields]) OR "EIA"[All Fields]) OR "CLIA"[All Fields]) OR "FIA"[All Fields]) OR "IFA"[All Fields]) OR "IgG"[All Fields])

For the PubMed search, the workflow is shown below:

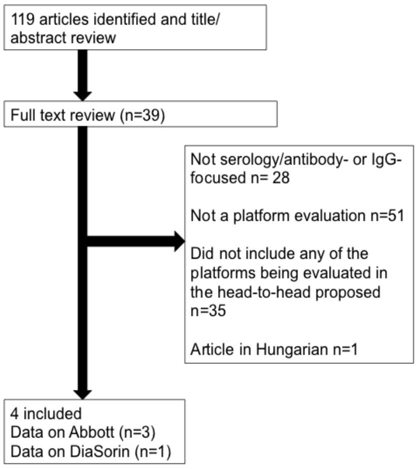


For the BioRxiv/MedRxiv searches, the workflow is shown below:

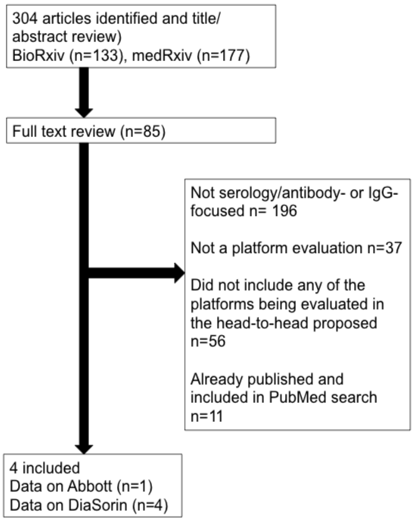


Article screening results can be found in Supplementary Dataset SD1.

**B. Cohorts from which samples were obtained**

'Known negative' samples

- Oxford BioBank, Oxfordshire, UK; Oxfordshire Clinical Research Ethics Committee 08/H0606/107+5

'Known positives' samples

- Gastro-intestinal illness in Oxford: COVID substudy [Sheffield REC, reference: 16/YH/0247]
- ISARIC/WHO Clinical Characterisation Protocol for Severe Emerging Infections [Oxford REC C, reference 13/SC/0149]
- Sepsis Immunomics project [Oxford REC C, reference:19/SC/0296])
- Volunteer plasma donors being screened for convalescent plasma studies by NHS Blood and Transplant (NHSBT; RECOVERY [Cambridge East REC (ref: 20/EE/0101)] and REMAP-CAP [EudraCT 2015-002340-14] studies).

**C. Oxford immunoassay (OIA) validation and calibration**

**Schematic of OIA and workflow**

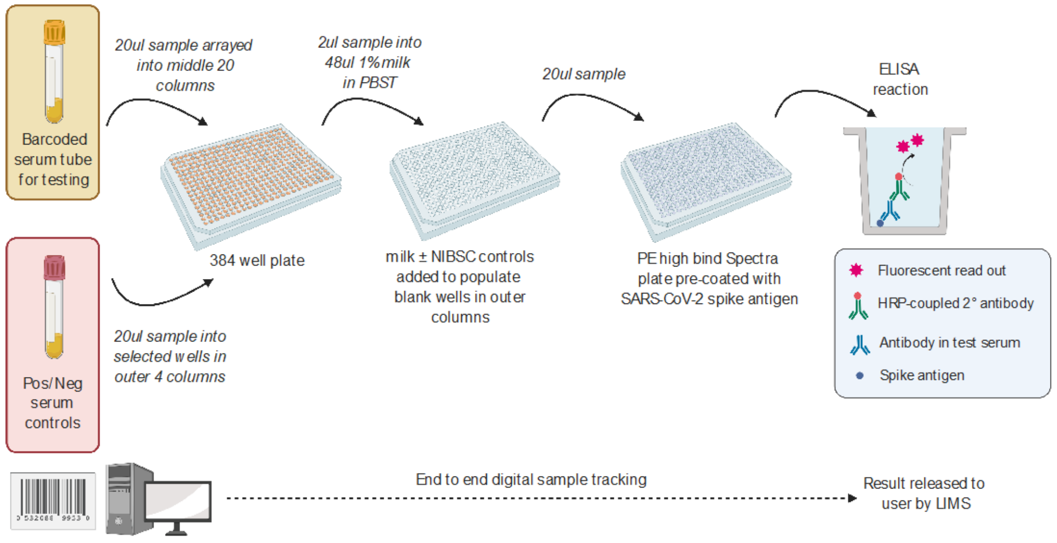


**Plate layout - schematic**

| | 1 | 2 | 3 | 4 | 5 | 6 | 7 | 8 | 9 | 10 | 11 | 12 | 13 | 14 | 15 | 16 | 17 | 18 | 19 | 20 | 21 | 22 | 23 | 24 |
|---|---|---|---|---|---|---|---|---|---|---|---|---|---|---|---|---|---|---|---|---|---|---|---|---|
| A | 3 | 1:400 | | | | | | | | | | | | | | | | | | | | | (±NIBSC) | 1:50 |
| B | 1 | 1:800 | | | | | | | | | | | | | | | | | | | | | 1:25 | 1:100 |
| C | 0.3 | 1:1600 | | | | | | | | | | | | | | | | | | | | | (±NIBSC) | 1:200 |
| D | 0.1 | 1:3200 | | | | | | | | | | | | | | | | | | | | | 1:25 | 1:400 |
| E | 0.03 | 1:400 | | | | | | | | | | | | | | | | | | | | | (±NIBSC) | 1:800 |
| G | 0.01 | 1:800 | | | | | | | | | | | | | | | | | | | | | 1:25 | 1:1600 |
| H | 0.003 | 1:1600 | | | | | | | | | | | | | | | | | | | | | (±NIBSC) | 1:50 |
| I | 0.001 | 1:3200 | | | | | | | | | | | | | | | | | | | | | 1:25 | 1:100 |
| J | | 3 | | | | | | | | | | | | | | | | | | | | | (±NIBSC) | 1:200 |
| K | | 1 | | | | | | | | | | | | | | | | | | | | | 1:25 | 1:400 |
| L | | 0.3 | | | | | | | | | | | | | | | | | | | | | 1:50 | 1:800 |
| M | | 0.1 | | | | | | | | | | | | | | | | | | | | | 1:25 | 1:1600 |
| N | | 0.03 | | | | | | | | | | | | | | | | | | | | | 1:200 | 1:3200 |
| O | | 0.01 | | | | | | | | | | | | | | | | | | | | | 1:25 | 1:50 |
| P | | 0.003 | | | | | | | | | | | | | | | | | | | | | 1:800 | 1:200 |
| Q | | 0.001 | | | | | | | | | | | | | | | | | | | | | 1:25 | 1:800 |

Samples

Milk controls (background)
Monoclonal antibody (CR3022) - serial dilution
NHSBT high plasma standard (donor id: 10062)
NHSBT medium plasma standard (donor id: 10061)
NHSBT low plasma standard (donor id: 10063)
Negative (pre-pandemic) control
NIBSC reagent (not always added)

**Assay modifications**

The assay was implemented as in (1), but with the following minor modifications:

- A 4-times instead of 5-times wash after incubating the sera
- Incubating with the secondary antibody for 90mins instead of 60 mins
- Adding a further 2-times wash with PBS after the secondary antibody had been washed away three times with PBS-T
- Incubating with 20µL of QuantaRed™ Enhanced Chemifluorescent HRP Substrate Kit (Thermo Scientific, Waltham Massachusetts, USA) for three instead of four minutes before the addition of 2µL of the stop solution

**Calibration of the OIA assay**

A total 8 milk 'blank' wells were included in each plate to determine a background reading which was subtracted from the raw signal per well to obtain a net reading.

A panel of dilution series made from the 3 NHSBT controls and the monoclonal antibody (CR3022), run on each 384 well plate, were used to calibrate readings between plates and batches. NHSBT 10061 was used at 1:25, 1:50, 1:100, 1:200, 1:400, 1:800, 1:1600, 1:3200, NHSBT 10062 at 1:25, 1:400, 1:800, 1:1600, 1:3200 and NHSBT at 1:25, 1:50, 1:100, 1:200, 1:400, 1:800, 1:1600 dilutions. CR3022 was used at concentrations of 3, 1, 0.3, 0.1, 0.03, 0.01, 0.003, 0.001 µg/ml. The majority of controls were included in duplicate (n=50 overall). An additional 5 NIBSC controls were run on a subset of runs. A single negative control, BD01 (a pre-pandemic blood donor serum provided by NHSBT) was also included on all plates.

A reference set of assay values for these controls were determined from data obtained on 3rd June 2020. All runs were converted into "3rd June" units using a natural cubic spline based linear regression model. For visualisation, values obtained for each control dilution within each 384 well plate were plotted on the x-axis and the reference values plotted on the y-axis. A linear regression model was fitted between the two, transforming the values on the x-axis using a 3-knot natural cubic spline (see Figure below). Model parameters were then used to convert net readings for all samples in the plate to normalised net readings.

**Example mapping between net readings and normalised reference readings for control sample dilutions.**

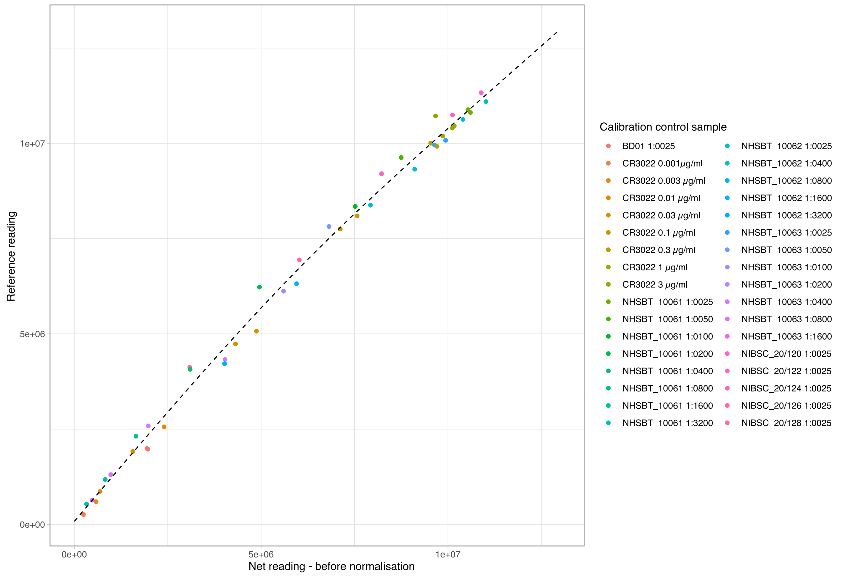


After initial model fitting a heuristic check for outliers was performed searching for differences between the normalised net readings and reference values >800,000 units. Such points were excluded, typically ≤2 out of a total of 50 controls, before re-fitting a final model.

**Derivation of OIA diagnostic thresholds**

We derived thresholds for the Oxford immunoassay (OIA) using an independent set of derivation samples:

- Known positives; defined by SARS-CoV-2 RT-PCR positive nose/throat swab; n=120
  - Acute; n=21
    - >10-28 days from symptom-onset; n=21
  - Convalescent; n=99 individuals

        ▪ >28 days from symptom-onset (from Oxfordshire); n=18
        ▪ >28 days from PCR-positive result (obtained from NHSBT); n=81 samples

- MERS-CoV anti-sera; n=1
- Other respiratory virus infections; n=23
    - Acute seasonal coronavirus-positive samples (≤28 days post-respiratory PCR [BioFire FilmArray RP panel]); n=6
    - Convalescent seasonal coronavirus-positive samples (>28 days post-respiratory PCR [BioFire FilmArray RP panel]; n=11
    - Acute non-coronavirus respiratory virus infections; n=6 (≤28 days post-respiratory PCR [BioFire FilmArray RP panel])
- Pre-pandemic controls n=1205
    - Serum samples taken from in- or outpatients having health monitoring, or samples as part of clinical management; n=954
    - Pre-pandemic blood donors recruited by NHSBT; n=251

We used a prespecified specificity target of 99% to set a threshold for determining a threshold for presence of antibody. The distribution of results obtained is shown in the figure below.

**OIA diagnostic threshold derivation samples**

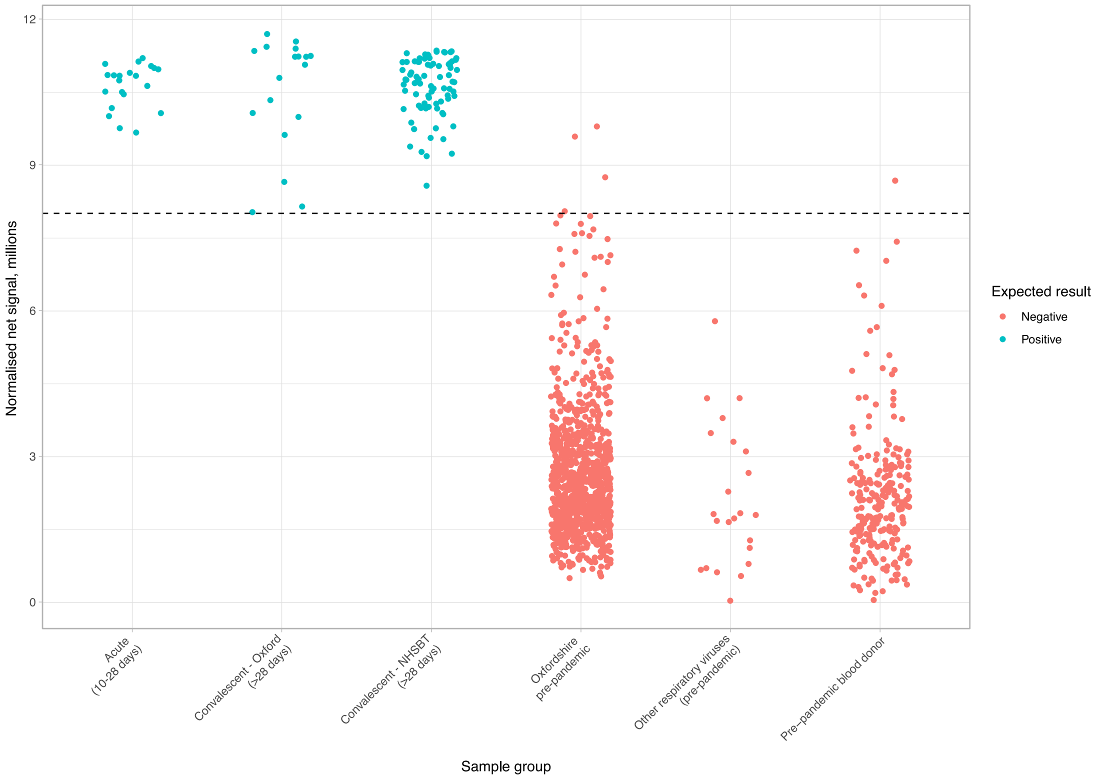


At ≥10 days post symptom onset, excluding the MERS anti-sera (which was positive) but including the 23 samples from individuals with other viral infections, derivation sensitivity and specificity (at a normalised threshold of 8 million units) were 100% (120/120; 95% CI: 97.0-100.0%) and 99.6 (1223/1228; 95% CI: 99.1-99.9%) respectively.

**Other methods**
Sensitivity was calculated using the following equation:
Number of true positive tests/(number of true positive tests+number of false negative tests)

Specificity was calculated using the following equation:
Number of true negative tests/(number of true negative tests+number of false positive tests)

The positive predictive value (PPV) was calculated from the following equation:
(Sensitivity*prevalence/[(sensitivity*prevalence)+(1-specificity)*(1-prevalence)]

The negative predictive value (NPV) was calculated from the following equation:
Specificity*(1-prevalence)/[(1-sensitivity)*prevalence+(specificity*(1-prevalence))]

## Supplementary Tables

**Table S1. Summary of serum/plasma samples used for head-to-head analysis of five immunoassays for the detection of SARS-CoV-2 antibodies.** This reflects the details for samples that were evaluated across all assays.

| Group | Source | Number of samples | Days from symptom onset, median (IQR; min, max; number of samples) | Days from PCR-positive test, median (IQR; min, max; number of samples) |
|---|---|---|---|---|
| Known negative | Healthy individuals 30-50 years of age, collected between 2015-2018 in Oxfordshire (Oxford BioBank, www.oxfordbiobank.org.uk) | 976 | n/a | n/a |
| Known positive | Healthcare workers and patients ≥18 years of age at Oxford University Hospital NHS Foundation Trust, Oxfordshire, UK | 158 | 36·5 (28-53; 20, 73; n=158) | 27 (4-58; 3, 59; n=105) |
| Known positive | Volunteer plasma donors ≥18 years of age via NHS Blood and Transplant (NHSBT), across the UK | 378 | All samples ≥28 days post-symptom onset* | 44 (40-49; 32, 82; n=378) |

* Although specific data on time from symptoms is not available for this group, all donors had to be have been at least 28 days post-symptom onset to be eligible for sampling: see https://www.nhsbt.nhs.uk/plasma-trial/

**Table S2. Summary of the commercial immunoassays evaluated.** Information presented is based on the product literature released by each manufacturer, using versions active on 8-June-2020 when our protocol was finalised.

| **Assay and analyser used** | **Viral target and antibody type** | **Sample type** | **Sensitivity (95% CI) on samples taken ≥14 days post-symptom onset/post-positive RT-PCR, number of samples** | **Specificity (95% CI), number of samples** | **Manufacturers' thresholds** |
|---|---|---|---|---|---|
| Abbott SARS-CoV-2 Immunoassay, Architect i2000SR | Nucleocapsid protein, IgG | Serum, serum separator tube and plasma (ACD, CPD, CPDA-1, dipotassium EDTA, tripotassium EDTA, lithium heparin, lithium heparin separator tube, sodium citrate, sodium heparin) | 96.77% (90.86, 99.33), 88<br>(≥14 days post-symptom onset) | 99.63% (99.05, 99.90), 1070 | Negative: <1.4<br>Positive: ≥1.4 |
| DiaSorin LIAISON® SARS-CoV-2 S1/S2 IgG, LIAISON® XL | Spike protein S1/S2, IgG | Serum, plasma (sodium heparin, lithium heparin, potassium EDTA) | 97.56% (87.40, 99.57), 14<br>(≥15 days from diagnosis (RT-PCR)) | 98.5% (97.6, 99.2) , 1090 | Negative: <12.0 AU/mL<br>Equivocal: 12.0 ≤ x <15.0 AU/mL<br>Positive: ≥15.0 AU/mL |
| Roche Elecsys® Anti-SARS-CoV-2, Cobas e 411 | Nucleocapsid protein, Total antibody | Serum collected using standard sampling tubes. Li-heparin, K2-EDTA and K3-EDTA plasma | 100% (88.1, 100), 29<br>(≥14 days from diagnosis (RT-PCR)) | 99.81% (99.65, 99.91), 5272 | Non-reactive: <1.0<br>Reactive: ≥1.0 |
| Siemens SARS-CoV-2 Total (COV2T), Atellica Solution immunoassay analyzer | Spike protein S1 RBD, Total antibody | Serum and plasma (potassium EDTA and lithium heparin) | 100.00% (91.59, 100.00), 42<br>(≥14 days from diagnosis (RT-PCR)) | 99.82 (99.34, 99.98), 1091 | Non-reactive: <1.0<br>Reactive: ≥1.0 |

**Table S3. Summary of external evaluations of assay specificity**

| Publication DOI/URL, first author, date | Cohort | Sample number | Reactive/ positive | Specificity % (95% CI) | Notes |
|---|---|---|---|---|---|
| Abbott | | | | | |
| 10.1128/JCM.01029-20, Brecher S et al 27/May/2020 | RT-PCR-negative for SARS-CoV-2, PCR (Respiratory Panel 2, Film Array, BioFire Diagnostics) positive for other seasonal coronaviruses.<br><br>Plasma samples taken >4 weeks after the positive respiratory PCR | 9 | 0 | 100% (66.3,100) | Very small study focused on analytical specificity in the context of SARS-CoV-negative (by RT-PCR) individuals.<br><br>Elderly male population as the study was undertaken in three regional Veterans Affairs (VA) institutions in the US. |
| 10.1093/clinchem/hvaa120 Tang MS et al, 18/Jun/2020 (as BioRxiv preprint on 10/May/2020 | Control specimens included:<br>80 patients symptomatic but PCR negative for SARS-CoV-2; 50 serum specimens collected and frozen in 2015 before the emergence of SARS-CoV-2; 5 specimens from patients with other coronaviruses confirmed by molecular testing but PCR negative for COVID-19 (including Coronaviruses HKU1, NL63, and 229E); 4 specimens from patients with Influenza A or B.<br>14 specimens with potentially interfering antibodies were also included: 5 were positive for CMV IgG, 3 were positive for EBV VCA IgG, 3 were positive for EBV VCA IgM, 2 were positive for both EBV VCA IgG and IgM, and 1 was positive for rheumatoid factor | 153 | 1 | 99.4% (96.41, 99.98) | The single false-positive was a patient with a consistent syndrome and "prolonged exposure to a family member with PCR confirmed COVID-19" i.e. a likely Covid-19 case that was falsely negative by RT-PCR, highlighting the difficulty with using post-pandemic samples as a "known negative" group for evaluating specificity. |
| 10.1128/JCM.00941-20, Bryan A et al, 7/May/2020 | Pre-pandemic specimens 2018-2019 sent to the clinical laboratory | 1020 | 1 | 99.9% (99.5, 100) | Proposed the use of AUC analysis to adjust thresholds: "These analyses indicated that optimal thresholds for the serologic diagnosis of SARS-CoV-2 was 1.42-1.49 at ≥ 17 days from symptom onset (sensitivity and specificity 100%); 0.7 at ≥ 14 days from onset (Sens 97.9%, Spec 99.6%); 0.7 at ≥ 10 days from onset (Sens 94.4%, Spec 99.6%); and 0.7 at ≥ 7 days from onset (Sens 88.0%, Spec 99.6%)"<br><br>Pre-pandemic negative group represented samples submitted from 1010 individuals for HSV Western blot serology evaluation - i.e. likely to represent a biased subset of the population in whom HSV testing was being performed. |

| | | | | | |
|---|---|---|---|---|---|
| https://doi.org/10.1101/2020.05.18.20101618, Jääskeläinen AJ et al, 22/May/2020 | Pre-pandemic samples from 2018-2019<br>81 from 81 individuals including:<br>39 samples had autoantibodies<br>3 acute EBV<br>4 acute coronavirus<br>35 from patients with respiratory infection | 81 | NA | 95.1% (NA) | Small sample size, with all samples coming from individuals with other infections/autoantibodies, confidence intervals not estimated. |
| https://assets.publishing.service.gov.uk/government/uploads/system/uploads/attachment_data/file/887221/PHE_Evaluation_of_Abbott_SARS_CoV_2_IgG.pdf PHE, 18/May/2020 | Total<br><br>Negative samples- historic samples (samples stored before mid-2019)<br><br>Confounder negative samples (rheumatoid factor, CMV, EBV or VZV positive)<br><br>Seasonal coronavirus positive samples | 757 samples (Unknown number of individuals)<br><br>395 samples<br><br>351 samples<br><br>11 samples | 0<br><br>0<br><br>0<br><br>0 | 100% (97.79-100)<br><br>100% (99.1- 100.0)<br><br>100%  (71.5- 100.0)<br><br>100% (99.0- 100.0) | Threshold originally incorrectly reported as an Index (S/C) of 1.0. Numbers of individuals not reported. |
| DiaSorin Liaison SARS-CoV-2 S1/S2 IgG | | | | | |
| 10.1515/cclm-2020-0594, Tré-Hardy M et al, 25/May/2020 | Total<br><br>Confounders: COVID-19-negative but who had other viral, bacterial, parasitic or autoimmune pathologies<br><br>Other coronavirus infection: COVID-19-negative patients but positive to another strain of coronavirus (NL63 strain and OC43 strain)<br><br>Healthy subjects | 81<br><br>73<br><br>2<br><br>6 | 0 | 100% (95, 100) | Proposed the use of ROC analysis to improve thresholds resulting in specificity of 99% (95% CI: 93%–100%) and sensitivity of 100% (95% CI: 92%–100%).<br><br>No breakdown by sub-group. |
| https://doi.org/10.1101/2020.05.19.105445, Bonelli, F et al, 20/May/2020 | Pre-pandemic serum samples (routine laboratory, 2011)<br><br>Individuals with other coronavirus infections<br><br>SARS-CoV-2 RT-PCR negative patients<br><br>SARS-CoV-2 microneutralisation antibody negative samples<br><br>All negatives | 1140<br><br>10<br><br>50<br><br>180<br><br>1380 | | 98.5% (97.6, 99.1)<br><br>98.1% (97.2, 98.8) | No breakdown by sub-group. |
| https://doi.org/10.1101/2020.05.18.20101618, Jääskeläinen AJ et al, 22/May/2020 | Pre-pandemic samples from 2018-2019<br>81 from 81 individuals including:<br>39 samples had autoantibodies<br>3 acute EBV<br>4 acute coronavirus<br>35 from patients with respiratory infection | 70 | NA | 94.9% (NA) | Small sample size, with all samples coming from individuals with other infections/autoantibodies, confidence intervals not estimated.<br><br>Unclear which subset of 70 samples were used to evaluate the DiaSorin assay (insufficient sample to test all samples across all immunoassays included in the evaluation) |
| https://doi.org/10.1101/202 | Total 191 individuals (negative by 3 sequential RT-PCR results) | n= ? negative | NA | 96.8 (89.0-99.6) | Number of samples and individuals tested not |

| | | | | | |
|---|---|---|---|---|---|
| 0.05.22.20106328, Plebani M et al, 26/May/2020 | <br><br><br><br><br><br><br><br>Blood donors from 2015<br><br>Patients with autoimmune disease<br><br>Healthcare workers | samples<br><br>131 (pos and neg) samples from 271 individuals<br><br>101 individuals<br>19 individuals<br>71 individuals | | | clear. Not specified which subset of positive/ negative individuals/samples used.<br><br>Using optimised thresholds: 88.9% (78.4-95.4) |
| https://doi.org/10.1101/2020.04.23.20077156, GeurtsvanKessel CH et al, 05/May/2020 | Serum and plasma from people exposed to a human coronavirus (HCoV-229E, NL63 or OC43), SARS, MERS, or with a range of other respiratory viruses, and patients with recent CMV, EBV or *M. pneumoniae* infection.<br>(69/147 negatives run on Liaison) | 69 | 1 | 98.55% (92.24, 99.93) | Evaluation on samples from patients with other infections only. |
| Roche Elecsys® | | | | | |
| https://assets.publishing.service.gov.uk/government/uploads/system/uploads/attachment_data/file/887222/PHE_Evaluation_of_Roche_Elecsys_anti_SARS_CoV_2.pdf, PHE, 18/May/2020 | Total<br><br>Confounder negative samples- from the Sero-Evaluation Unit (SEU), Manchester that are rheumatoid factor (12 samples), CMV (6 samples), EBV (19 samples) or VZV (13 samples) positive. All but one were negative using the EuroImmun IgG assay<br><br>Porton negative samples- from the RIPL 2015 Lyme disease negative sample collection<br><br>Manchester negative samples- historic samples from the Seroepidemiology Unit (SEU) | 472 samples<br><br>50 samples<br><br><br><br><br><br><br><br>35 samples<br><br><br><br>387 samples | 0<br><br>0<br><br><br><br><br><br><br><br>0<br><br><br><br>0 | 100% (99.1-100)<br><br>Not reported individually<br><br><br><br><br><br><br>Not reported individually<br><br><br>100% (99.1- 100.0) | <br><br>100% (95.8- 100.0) for Confounder and Porton sample sets (n=85) combined; results for individual sub-groups not reported individually |
| Siemens | | | | | |
| No additional published data available at the time of review | | | | | |

**Table S4. Summary of external evaluations of assay sensitivity**

| ublication DOI/URL, first author, date | Cohort | Sample number | Reactive/ positive | Sensitivity (95% CI) | Notes |
|---|---|---|---|---|---|
| Abbott | | | | | |
| 10.1093/clinchem/hvaa120 Tang MS et al, 18/Jun/2020 (as BioRxiv preprint on 10/May/2020 | <br><br><br><br>Post-symptom onset:<br><3 days post-symptom onset<br><br>3-7 days post-symptom onset<br><br>8-13 days post-symptom onset<br><br>≥14 days post-symptom onset<br><br>Post-positive PCR:<br><3 days post-positive PCR<br><br>3-7 days post-positive PCR<br><br>8-13 days post-positive PCR<br><br>≥14 days post-positive PCR | 103 specimens from 48 individuals<br><br><br>12<br><br>6<br><br>23<br><br>48<br><br><br>42<br><br>22<br><br>23<br><br>16 | <br><br><br><br><br>0<br><br>20<br><br>11<br><br>45<br><br><br>20<br><br>13<br><br>16<br><br>13 | <br><br><br><br><br>0% (0.00, 26.47)<br><br>30.0% (11.89, 54.28)<br><br>47.8% (26.82, 69.41)<br><br>93.8 (82.80, 98.69%)<br><br><br>47.6% (32.0, 63.6)<br><br>59.1% (36.3, 79.3)<br><br>69.6% (47.1, 86.8)<br><br>81.3% (54.4, 96.0) | Multiple samples from individuals included, which may not accurately reflect intra-individual variation. Longitudinal samples from individuals used.<br><br>Symptom onset "subjectively reported by physicians" and retrospectively retrieved from case records, which the authors conclude may have affected the accurate estimation of disease onset in the study. |
| 10.1128/JCM.00941-20, Bryan A et al, 7/May/2020 | RT-PCR positive, March-April 2020<br><br><br>Post-symptom onset:<br>≤7 days post-symptom onset<br><br>8-10 days post-symptom onset<br><br>11-14 days post-symptom onset<br><br>15-17 days post-symptom onset<br><br>Post-positive PCR:<br>≤7 days post-positive PCR<br><br>8-10 days post-positive PCR<br><br>11-14 days post-positive PCR<br><br>15-17 days post-positive PCR | 680 specimens from 125 individuals | | <br><br><br><br>53.1% (39.4, 66.3)<br><br>82.4% (51.0, 76.4)<br><br>96.9% (89.5, 99.5)<br><br>100% (95.1, 100)<br><br><br>88.7% (78.5, 94.4)<br><br>97.2% (90.4, 99.5)<br><br>100.0% (95.4, 100.0)<br><br>100.0% (95.5, 100.0) | Proposed the use of AUC analysis to adjust thresholds:<br>"These analyses indicated that optimal thresholds for the serologic diagnosis of SARS-CoV-2 was 1.42-1.49 at ≥ 17 days from symptom onset (sensitivity and specificity 100%); 0.7 at ≥ 14 days from onset (Sens 97.9%, Spec 99.6%); 0.7 at ≥ 10 days from onset (Sens 94.4%, Spec 99.6%); and 0.7 at ≥ 7 days from onset (Sens 88.0%, Spec 99.6%)"<br><br>Elderly patient cohort (65% >60 years of age), with limited capacity to accurately ascertain symptom onset. Longitudinal samples from the same individuals included |
| https://doi.org/10.1101/2020.05.18.20101618, Jääskeläinen AJ et al, 22/May/2020 | RT-PCR positive by one of three methods: "...cobas® SARS-CoV-2 test on the Cobas® 6800 system (Roche Diagnostics, Basel, Switzerland), Amplidiag® COVID-19 test (Mobidiag, Espoo, Finland) and a protocol based on Corman et al (2020)." | 70 samples from 62 individuals | | 80.5% (NA) | Longitudinal samples from the same individuals included, small sample sizes. |
| [https://assets.publishing.service.gov.uk/government/uploads/syste](https://assets.publishing.service.gov.uk/government/uploads/syste) | RT-PCR positive, otherwise healthy individuals, "14 had an onset date ≤14 days prior to sample collection and 81 had an onset date ≥14 days prior to | 96 samples, unknown number of individuals | | | It should be noted here that none of the patients with previously positive PCR who tested negative by this assay had been |

| | | | | | |
|---|---|---|---|---|---|
| m/uploads/attachment_data/file/887221/PHE_Evaluation_of_Abbott_SARS_CoV_2_IgG.pdf<br>PHE,<br>18/May/2020 | sample collection.” (number quoted in text different from that in table)<br><br>Hospital admission to sample date <=10<br><br>Asymptomatic<br><br>Reported symptom onset to sample date<br>11-20 days<br><br>21-30 days<br><br>31-40 days<br><br>41-50 days<br><br>From 14 days<br><br>From 21 days | 14<br><br>1<br><br>5<br><br>31<br><br>37<br><br>8<br><br>82<br><br>76 | 10<br><br>1<br><br>5<br><br>29<br><br>35<br><br>7<br><br>77<br><br>71 | 71.4% (41.9, 91.6)<br><br>100% (2.5, 100)<br><br>100% (47.8-100)<br><br>93.6% (78.6-99.2)<br><br>94.6% (81.8-99.3)<br><br>87.5% (47.3-99.7)<br><br>93.9% (86.3-98.0)<br><br>93.4% (85.3-97.8) | hospitalised for COVID-19 disease and most likely had a mild disease outcome.<br><br>NB reported sensitivities for 14/21 days were switched in table/conclusion.<br><br>On the left is data from Table 5.<br>Conclusion states: “93.4% (95%CI 85.3-97.8) for samples collected ≥14 days post symptom onset<br>93.9% (95%CI 86.3-98.0) for samples collected ≥21 days post symptom onset.” |
| DiaSorin Liaison SARS-CoV-2 S1/S2 IgG | | | | | |
| 10.1515/cclm-2020-0594,<br>Tré-Hardy M et al,<br>25/May/2020 | RT-PCR-positive patients with mild, moderate, severe and critical infection based on CT appearances and clinical symptoms<br><br>Delay between onset of symptoms and RT-PCR was 4 days (±1 day)<br><br>Blood sample “2 weeks after the RT-qPCR positive detection, and according to the manufacturer’s cut-off”<br><br>No information on RT-PCR test used | 44 | 40 | 91% (79, 96) | Proposed the use of ROC analysis to improve thresholds resulting in specificity of 99% (95% CI: 93%–100%) and sensitivity of 100% (95% CI: 92%–100%) |
| https://doi.org/10.1101/2020.05.19.105445,<br>Bonelli, F et al,<br>20/May/2020 | RT-PCR positive patients with moderate/severe disease (admission/discharge samples)<br><br>Microneutralisation antibody positive samples<br><br>≤5 days post RT-PCR-positive<br><br>>5 days post-RT-PCR-positive | 105 samples, unknown number of individuals<br><br>124 | | 22.6% (14.2, 33.0)<br><br>88.2% (81.3, 93.2) | Unknown number of individuals tested. |
| https://doi.org/10.1101/2020.05.18.20101618,<br>Plebani M et al,<br>26/May/2020 | RT-PCR positive by one of three methods: “...cobas® SARS-CoV-2 test on the Cobas® 6800 system (Roche Diagnostics, Basel, Switzerland), Amplidiag® COVID-19 test (Mobidiag, Espoo, Finland) and a protocol based on Corman et al (2020).” | 70 samples from 62 individuals | | 43.8% (NA) | |
| https://doi.org/10.1101/2020.05.22.20106328,<br>Jääskeläinen AJ et al,<br>22/May/2020 | 80 with at least one positive nasopharyngeal swab, consisting of:<br><br>16 healthcare workers, not hospitalised, SARS-CoV-2 positive | n= ?<br><br>131 samples from 271 individuals run on Liaison in total | | Sens 82.4% (71.2, 90.5) | Using optimised thresholds: Sens 97.1% (89.8, 99.6)<br><br>Unclear which subset of positive/negative samples used for the evaluation |

| | | | | | |
|---|---|---|---|---|---|
| | 32 hospitalised SARS-CoV-2 patients with moderate disease (not requiring ventilation)<br><br>32 hospitalised SARS-CoV-2 patients with severe disease (requiring ventilation) | | | | |
| https://doi.org/10.1101/2020.04.23.20077156, GeurtsvanKessel CH et al, 05/May/2020 | RT-PCR confirmed COVID-19 patients with different levels of disease severity<br><br>All Severe<br><br>>14 days post symptoms | 53 samples, number individuals unknown.<br><br>53<br><br>18 | <br><br>39<br><br>17 | <br><br>73.58% (60.42, 83.56)<br><br>94.44% (74.24, 99.72) | Number of individuals not stated, all were from patients with severe disease. |
| https://www.fda.gov/medical-devices/emergency-situations-medical-devices/eua-authorized-serology-test-performance Accessed 3/6/20 | Included here as data different from product literature above. | 41 samples | 40 | 97.6% (87.4, 99.6) | Different sensitivity reported from that of the manufacturer; provenance of the results reported here unclear. |
| Roche Elecsys® | | | | | |
| https://assets.publishing.service.gov.uk/government/uploads/system/uploads/attachment_data/file/887222/PHE_Evaluation_of_Roche_Elecsys_anti_SARS_CoV_2.pdf PHE, 18/May/2020 | RT-PCR positive from a swab sample<br><br>Reported symptom onset to sample date<br>11-20 days<br>21-30 days<br>31-40 days<br>41-50 days<br><br>From 14 days<br>From 21 days<br><br>Hospital admission to sample date<br><=10 days<br><br>Asymptomatic, not admitted | 93 samples, number of individuals unknown<br><br>4<br>35<br>30<br>8<br><br>77<br>73<br><br>14<br><br>2 | 78<br><br>3<br>28<br>28<br>8<br><br>67<br>64<br><br>10<br><br>1 | 83.9% (74.8-90.7)<br><br>75.0% (19.4-99.4)<br>80.0% (63.1-91.6)<br>93.3% (77.9-99.2)<br>100.0% (63.1-100)<br><br>87.0% (77.4-93.6)<br>87.7% (77.9-94.2)<br><br>71.4% (41.9-91.6)<br><br>50.0% (1.3-98.7) | Number of individuals not reported.<br><br>Small numbers in sub-groups. |
| Siemens | | | | | |
| No additional published data available at the time of review | | | | | |

**Table S5. Positive and negative predictive values (PPV, NPV) for each assay, using the manufacturer's threshold and sensitivity and specificity for samples tested at ≥20 days, at population prevalences of 5%, 10%, 20% and 50%.** Absolute numbers of false negative (FN) and false positive (FP) per million tests are also shown.

| | **Population prevalence** | **Specificity (95% CI)** | **Sensitivity (95% CI)** | **FN per 1m tests (95% CI)** | **FP per 1m tests (95% CI)** | **Total errors per 1m tests (95% CI)** | **PPV** | **NPV** |
|---|---|---|---|---|---|---|---|---|
| Abbott | 5% | 99·9 (99·4-100) | 92·7 (90·2-94·8) | 3650 (3533-3770) | 950 (891-1012) | 4600 (4468-4735) | 98.0% | 99.6% |
| DiaSorin | 5% | 98·9 (98·0-99·4) | 96·2 (94·2-97·7) | 1900 (1817-1987) | 10450 (10252-10651) | 12350 (12135-12568) | 82.2% | 99.8% |
| Oxford | 5% | 99 (98·1-99·5) | 99·1 (97·8-99·7) | 450 (409-494) | 9500 (9311-962) | 9950 (9756-10147) | 83.9% | 99.95% |
| Roche | 5% | 99·8 (99·3-100) | 97·2 (95·4-98·4) | 1400 (1328-1475) | 1900 (1816-1987) | 3300 (3189-3414) | 96.2% | 99.85% |
| Siemens | 5% | 99·9 (99·4-100) | 98·1 (96·6-99·1) | 950 (891-1012) | 950 (891-1012) | 1900 (1816-1987) | 98.1% | 99.90% |
| | | | | | | | | |
| Abbott | 10% | 99·9 (99·4-100) | 92·7 (90·2-94·8) | 7300 (7134-7469) | 900 (842-961) | 8200 (8024-8379) | 99.0% | 99.2% |
| DiaSorin | 10% | 98·9 (98·0-99·4) | 96.2 (94·2-97·7) | 3800 (3680-3923) | 9900 (9707-10096) | 13700 (13473-13930) | 90.7% | 99.57% |
| Oxford | 10% | 99 (98·1-99·5) | 99·1 (97·8-99·7) | 900 (842-961) | 9000 (8158-9187) | 9900 (9707-10100) | 91.7% | 99.90% |
| Roche | 10% | 99·8 (99·3-100) | 97·2 (95·4-98·4) | 2800 (2697-2906) | 1800 (1718-1885) | 4600 (4468-4735) | 98.2% | 99.69% |
| Siemens | 10% | 99·9 (99·4-100) | 98·1 (96·6-99·1) | 1900 (1816-1987) | 900 (842-961) | 2800 (2697-2906) | 99.1% | 99.79% |
| | | | | | | | | |
| Abbott | 20% | 99·9 (99·4-100) | 92·7 (90·2-94·8) | 14600 (14366-14837) | 800 (746-857) | 15400 (15160-15643) | 99.6% | 98.2% |
| DiaSorin | 20% | 98·9 (98·0-99·4) | 96·2 (94·2-97·7) | 7600 (7431-7772) | 8800 (8618-8985) | 16400 (16152-16651) | 95.6% | 99.05% |
| Oxford | 20% | 99 (98·1-99·5) | 99·1 (97·8-99·7) | 1800 (1718-1885) | 8000 (7826-8177) | 9800 (9608-9995) | 96.1% | 99.77% |
| Roche | 20% | 99·8 (99·3-100) | 97·2 (95·4-98·4) | 5600 (5455-5748) | 1600 (1523-1680) | 7200 (7035-7368) | 99.2% | 99.30% |
| Siemens | 20% | 99·9 (99·4-100) | 98·1 (96·6-99·1) | 3800 (3680-3923) | 800 (746-857) | 4600 (4468-4735) | 99.6% | 99.53% |
| | | | | | | | | |
| Abbott | 50% | 99·9 (99·4-100) | 92·7 (90·2-94·8) | 36500 (36133-36894) | 500 (457-546) | 37000 (36631-37372) | 99.9% | 93.2% |
| DiaSorin | 50% | 98·9 (98·0-99·4) | 95 (94·2-97·7) | 19000 (18733-19270) | 5500 (5356-5647) | 24500 (24198-24805) | 98.9% | 96.30% |
| Oxford | 50% | 99 (98·1-99·5) | 99·1 (97·8-99·7) | 4500 (4370-4633) | 5000 (4863-5140) | 9500 (9311-962) | 99.0% | 99.10% |
| Roche | 50% | 99·8 (99·3-100) | 97·2 (95·4-98·4) | 14000 (13771-14232) | 1000 (939-1064) | 15000 (14763-15240) | 99.8% | 97.27% |
| Siemens | 50% | 99·9 (99·4-100) | 98·1 (96·6-99·1) | 9500 (9311-962) | 500 (457-546) | 10000 (9806-10197) | 99.9% | 98.13% |

**Table S6. Summary of concordance/discordance of results between assays for “known positive” samples analysed as part of the main analysis.** “+” denotes a positive result, “-” a negative result and “+/-“ an equivocal result (the latter relevant for the DiaSorin assay only). These data represent last samples per patient.

| Abbott | DiaSorin | OIA | Roche | Siemens | n | Acute samples (<20 days post symptom onset) | % Acute | Samples from PCR-positive cases (≥20 days post symptom onset) | % Positive | Pre-pandemic negative | % Negative |
|---|---|---|---|---|---|---|---|---|---|---|---|
| - | - | - | - | - | 977 | 25 | 2.6 | 3 | 0.3 | 949 | 97.1 |
| + | + | + | + | + | 551 | 62 | 11.3 | 489 | 88.7 | 0 | 0.0 |
| - | - | + | - | - | 19 | 5 | 26.3 | 4 | 21.1 | 10 | 52.6 |
| - | + | + | + | + | 18 | 2 | 11.1 | 16 | 88.9 | 0 | 0.0 |
| + | - | + | + | + | 12 | 6 | 50.0 | 6 | 50.0 | 0 | 0.0 |
| - | + | - | - | - | 11 | 0 | 0.0 | 0 | 0.0 | 11 | 100.0 |
| - | + | + | - | + | 10 | 4 | 40.0 | 6 | 60.0 | 0 | 0.0 |
| - | - | - | + | - | 5 | 2 | 40.0 | 1 | 20.0 | 2 | 40.0 |
| - | - | + | - | + | 5 | 4 | 80.0 | 1 | 20.0 | 0 | 0.0 |
| - | +/- | + | + | + | 5 | 1 | 20.0 | 4 | 80.0 | 0 | 0.0 |
| + | - | + | + | - | 4 | 3 | 75.0 | 1 | 25.0 | 0 | 0.0 |
| + | +/- | + | + | + | 4 | 1 | 25.0 | 3 | 75.0 | 0 | 0.0 |
| - | - | + | + | + | 3 | 1 | 33.3 | 2 | 66.7 | 0 | 0.0 |
| - | - | - | - | + | 2 | 0 | 0.0 | 1 | 50.0 | 1 | 50.0 |
| - | +/- | - | - | - | 2 | 0 | 0.0 | 0 | 0.0 | 2 | 100.0 |
| + | - | + | - | + | 2 | 2 | 100.0 | 0 | 0.0 | 0 | 0.0 |
| - | - | + | + | - | 1 | 0 | 0.0 | 1 | 100.0 | 0 | 0.0 |
| - | +/- | + | - | + | 1 | 1 | 100.0 | 0 | 0.0 | 0 | 0.0 |
| + | - | - | - | - | 1 | 0 | 0.0 | 0 | 0.0 | 1 | 100.0 |
| + | - | - | + | - | 1 | 1 | 100.0 | 0 | 0.0 | 0 | 0.0 |
| + | - | - | + | + | 1 | 1 | 100.0 | 0 | 0.0 | 0 | 0.0 |
| + | - | + | - | - | 1 | 1 | 100.0 | 0 | 0.0 | 0 | 0.0 |
| + | + | + | - | + | 1 | 1 | 100.0 | 0 | 0.0 | 0 | 0.0 |
| + | +/- | + | - | + | 1 | 1 | 100.0 | 0 | 0.0 | 0 | 0.0 |

**Table S7. Summary of concordance/discordance of results between assays for early acute samples analysed at <14 days post-symptom onset.** First sample per patient analysed; total n=118.

| Abbott | DiaSorin | OIA | Roche | Siemens | Acute samples (<14 days post symptom onset) | % of samples |
|---|---|---|---|---|---|---|
| - | - | - | - | - | 32 | 27 |
| + | + | + | + | + | 40 | 34 |
| - | - | + | - | - | 6 | 5 |
| - | + | + | + | + | 3 | 3 |
| + | - | + | + | + | 11 | 9 |
| - | + | - | - | - | 0 | 0 |
| - | + | + | - | + | 5 | 4 |
| - | - | - | + | - | 2 | 1.7 |
| - | - | + | - | + | 5 | 4 |
| - | +/- | + | + | + | 1 | 0.8 |
| + | - | + | + | - | 3 | 0 |
| + | +/- | + | + | + | 1 | 0.8 |
| - | - | + | + | + | 1 | 0.8 |
| - | - | - | - | + | 0 | 0 |
| - | +/- | - | - | - | 0 | 0 |
| + | - | + | - | + | 3 | 3 |
| - | - | + | + | - | 0 | 0 |
| - | +/- | + | - | + | 0 | 0 |
| + | - | - | - | - | 0 | 0 |
| + | - | - | + | - | 1 | 0 |
| + | - | - | + | + | 2 | 1.7 |
| + | - | + | - | - | 1 | 0.8 |
| + | + | + | - | + | 0 | 0 |
| + | +/- | + | - | + | 1 | 0.8 |

**Figure S1. Sample collections and inclusions/exclusions.** For de-duplication of samples by individual, the latest sample meeting the MHRA criteria (i.e. latest sample taken ≥20 post-symptom onset) was included. The Table below the figure summarises the partial results for the five samples that were of insufficient volume to run across all platforms, and the text below the table the results for the 18 samples that did not pass QC due to liquid handling failures for the OIA.

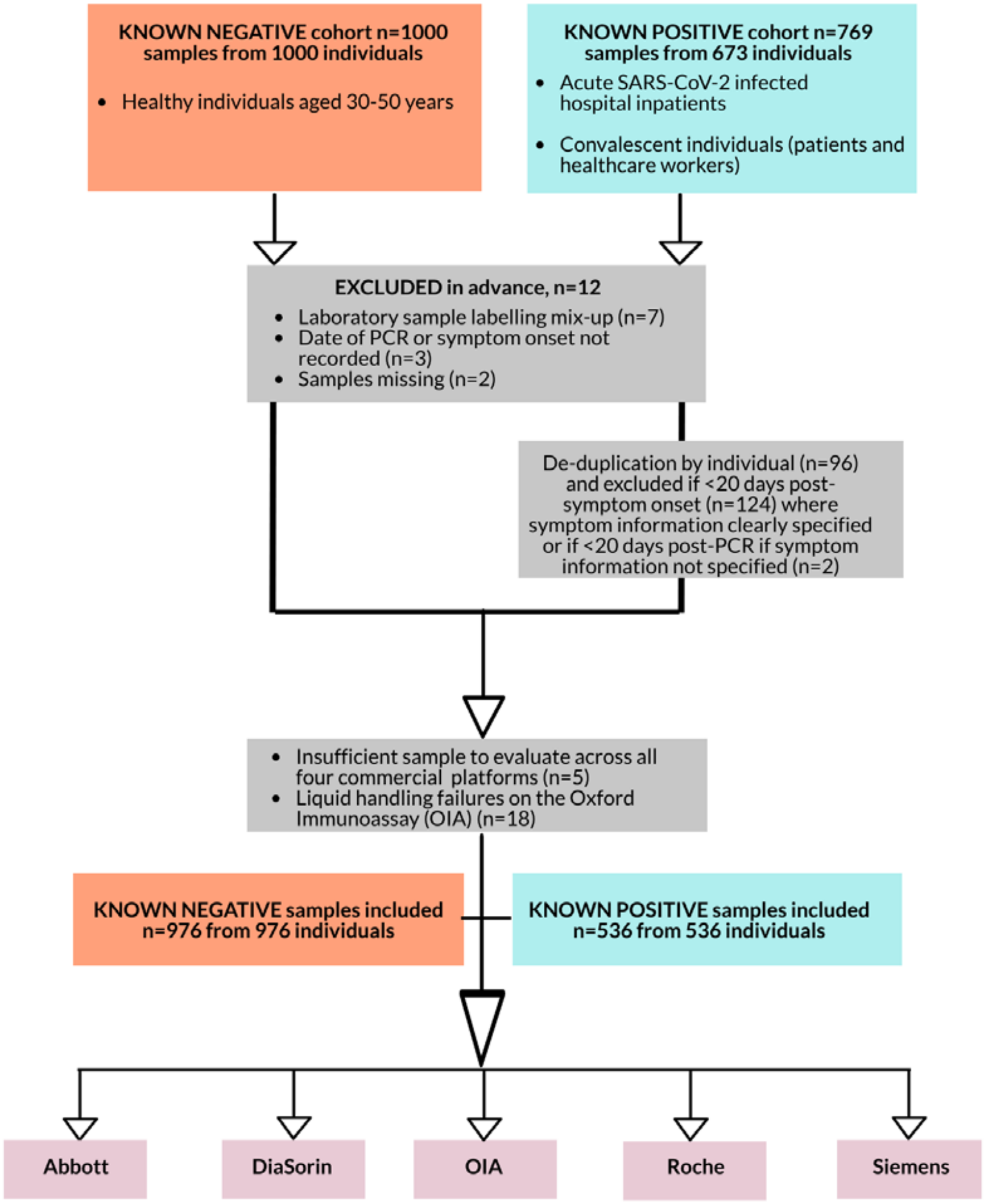


**Partial results for five samples for which there was insufficient sample to run across all four platforms**

| Sample barcode | Expected result | Days since symptom onset | Platform | Actual result |
|---|---|---|---|---|
| 900753 | Negative | n/a | Abbott | Negative |
| | | | DiaSorin | Negative |
| | | | Roche | Negative |
| 500379 | Positive | 40 | Abbott | Positive |
| | | | DiaSorin | Positive |
| 500380 | Positive | 41 | Abbott | Positive |
| | | | DiaSorin | Positive |
| 500381 | Positive | 41 | Abbott | Negative |
| | | | DiaSorin | Negative |
| 500384 | Positive | 42 | Abbott | Positive |
| | | | DiaSorin | Positive |

For the 18 samples that failed on the OIA, all were in the pre-pandemic (known negative) sample group, and all were negative by Abbott, Roche and Siemens; a single sample was positive on the DiaSorin assay (900079).

**Figure S2. Sensitivity and specificity (95% confidence intervals) plotted for each assay on all samples ≥20 days post-symptom onset in confirmed laboratory cases of SARS-CoV-2 for positive cases, and >6 months prior to the first known COVID-19 cases for negatives.** A target performance in line with the UK MHRA Target Product Profile is shown (dashed line) including the required lower bound of the 95% confidence interval (dotted line) for both sensitivity and specificity. Data are presented for all known negative samples (Abbott n=995, DiaSorin n=995, OIA n=977, Roche n=995, Siemens n=994) and all known positive samples run across (Abbott n=540, DiaSorin n=540, OIA n=540, Roche n=536, Siemens n=536) assays; equivocal results were excluded from the calculation of sensitivity and specificity for the DiaSorin assay (n=9).

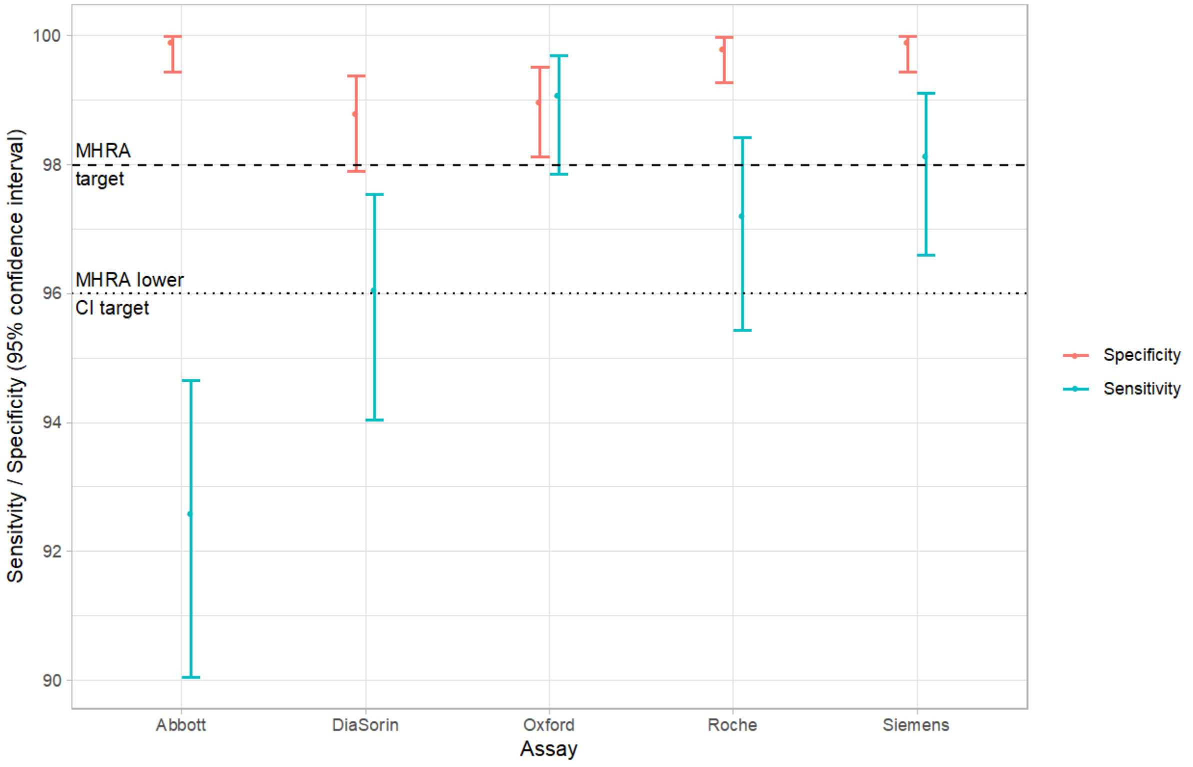

**Figure S3. Distribution of numerical results obtained for each commercial assay on all samples taken ≥20 days post-symptom onset (i.e. not restricted to samples only run across all platforms).** Results are represented as A. histograms, to enable assessment of the frequency of values, and B. dotplots, to review scatter of values, especially around thresholds. Pre-specified assay thresholds are shown as dashed lines. For the purposes of plotting values on a log scale, values of zero were set to the lowest non-zero value and results of greater or less than the largest or smallest values were truncated to the largest and smallest values. Data are presented for all known negative samples (Abbott n=995, DiaSorin n=995, OIA n=977, Roche n=995, Siemens n=994) and all known positive samples run across (Abbott n=540, DiaSorin n=540, OIA n=540, Roche n=536, Siemens n=536) assays.

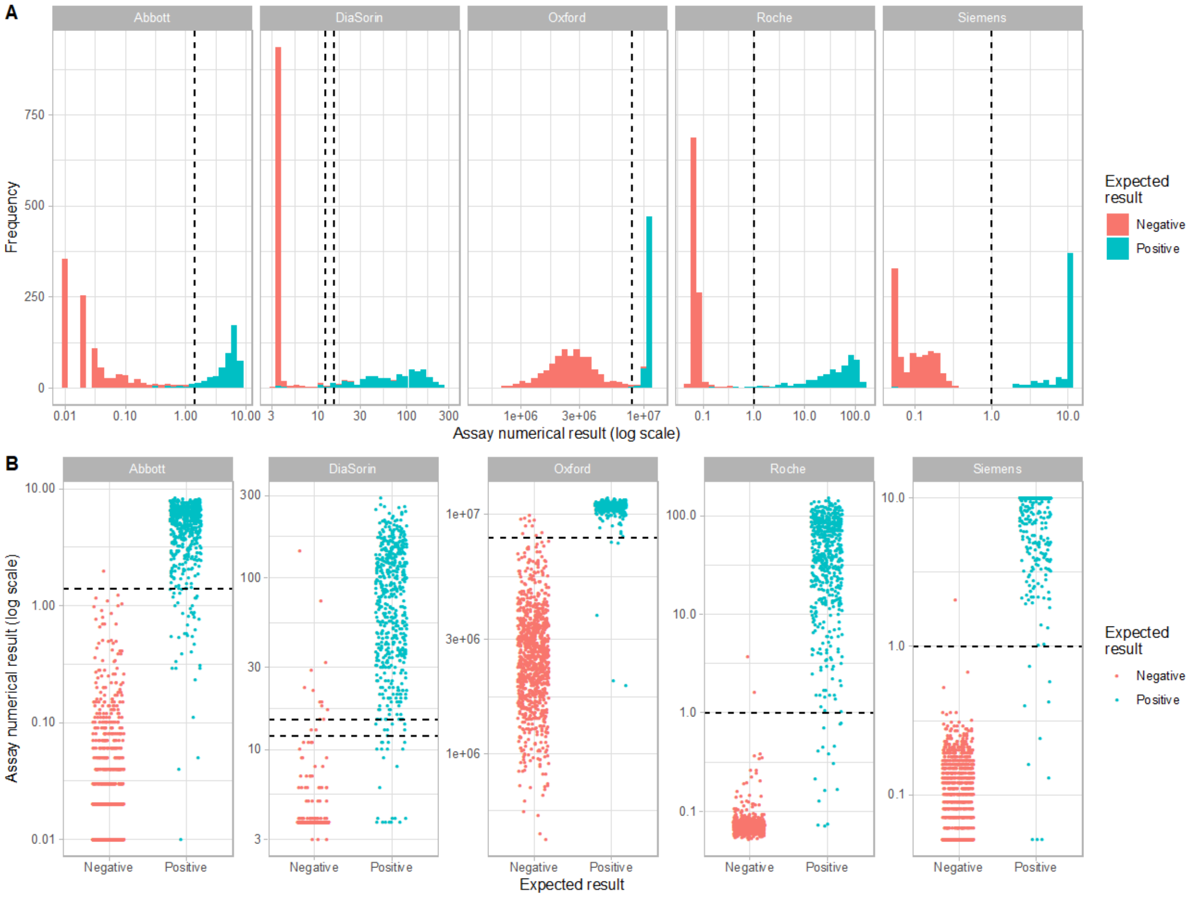

**Figure S4. Sensitivity and specificity (95% confidence intervals) plotted for each assay on samples ≥14 days post-symptom onset in confirmed laboratory cases of SARS-CoV-2 for positive cases, and >6 months prior to the first known COVID-19 cases for negatives.** A target performance in line with the UK MHRA Target Product Profile is shown (dashed line) including the required lower bound of the 95% confidence interval (dotted line) for both sensitivity and specificity. Data are presented for samples run across all platforms (n=976 and 561 for pre-pandemic and samples from positive cases respectively).

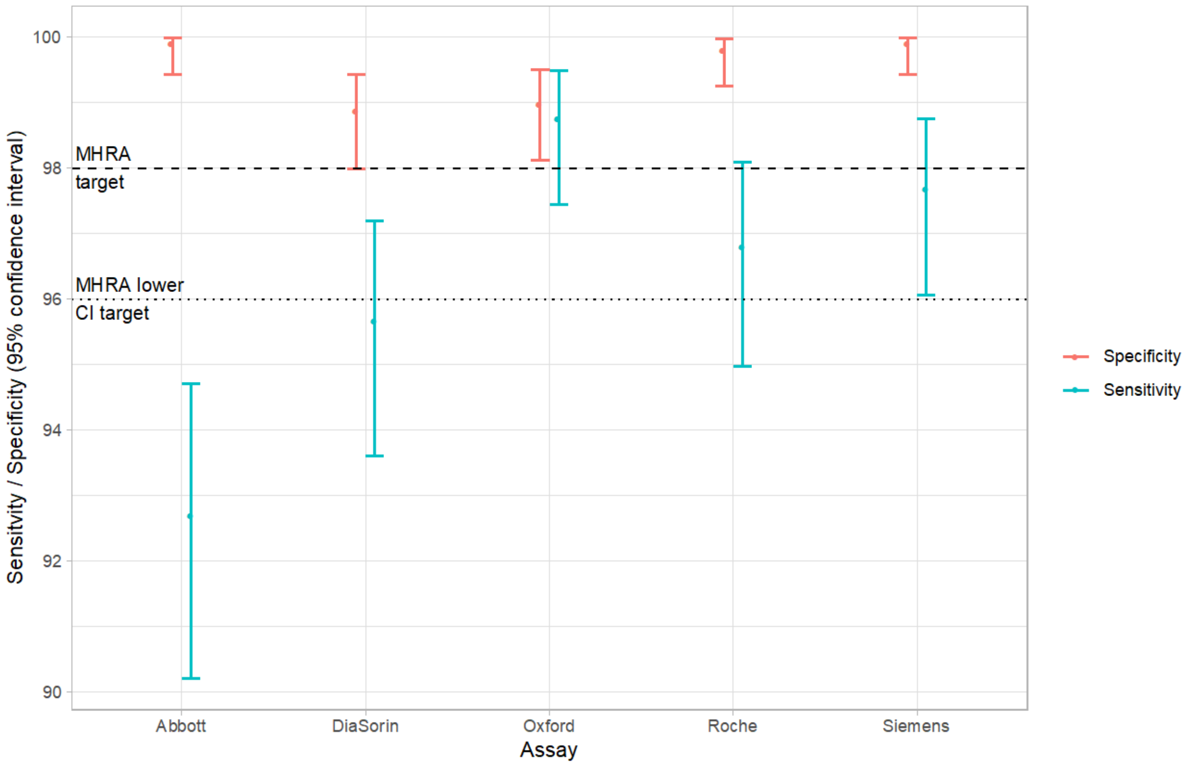

**Figure S5. Sensitivity and specificity (95% confidence intervals) plotted for each assay on samples ≥30 days post-symptom onset in confirmed laboratory cases of SARS-CoV-2 for positive cases, and >6 months prior to the first known COVID-19 cases for negatives.** A target performance in line with the UK MHRA Target Product Profile is shown (dashed line) including the required lower bound of the 95% confidence interval (dotted line) for both sensitivity and specificity. Data are presented for samples run across all platforms (n=976 and n=490 for pre-pandemic and samples from positive cases respectively).

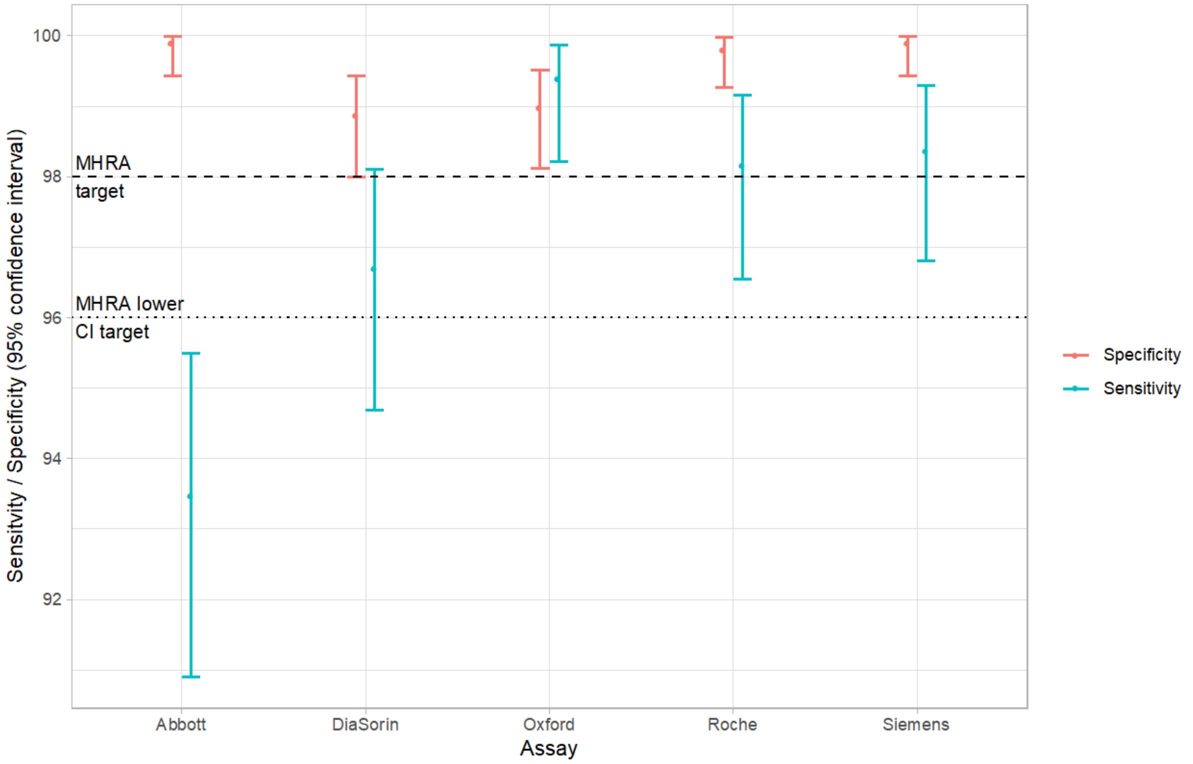

**Figure S6. ROC curves for each assay on samples taken ≥14 days after the onset of symptoms.** The green shaded area represents sensitivity and specificity of ≥98% and ≥98% respectively. Assay values associated with 10 exemplar points on the ROC curve are shown in each panel. Data are presented for 976 known negative samples and 561 known positive samples run on each assay.

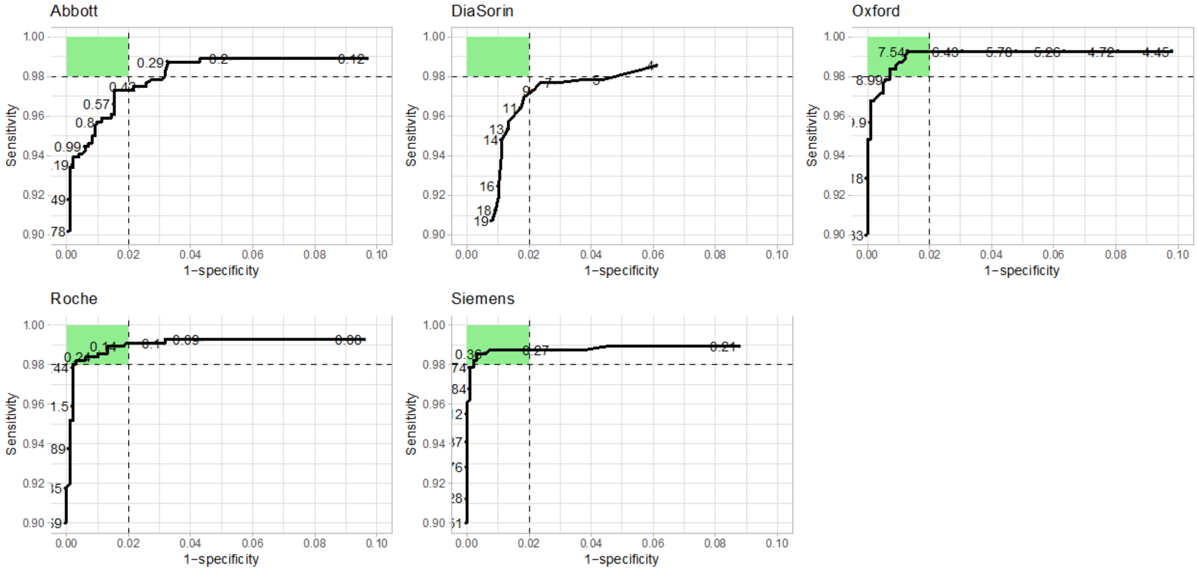

**Figure S7. ROC curves for each assay on samples taken ≥30 days after the onset of symptoms.** The green shaded area represents sensitivity and specificity of ≥98% and ≥98% respectively. Assay values associated with 10 exemplar points on the ROC curve are shown in each panel. Data are presented for 976 known negative samples and 490 known positive samples run on each assay.

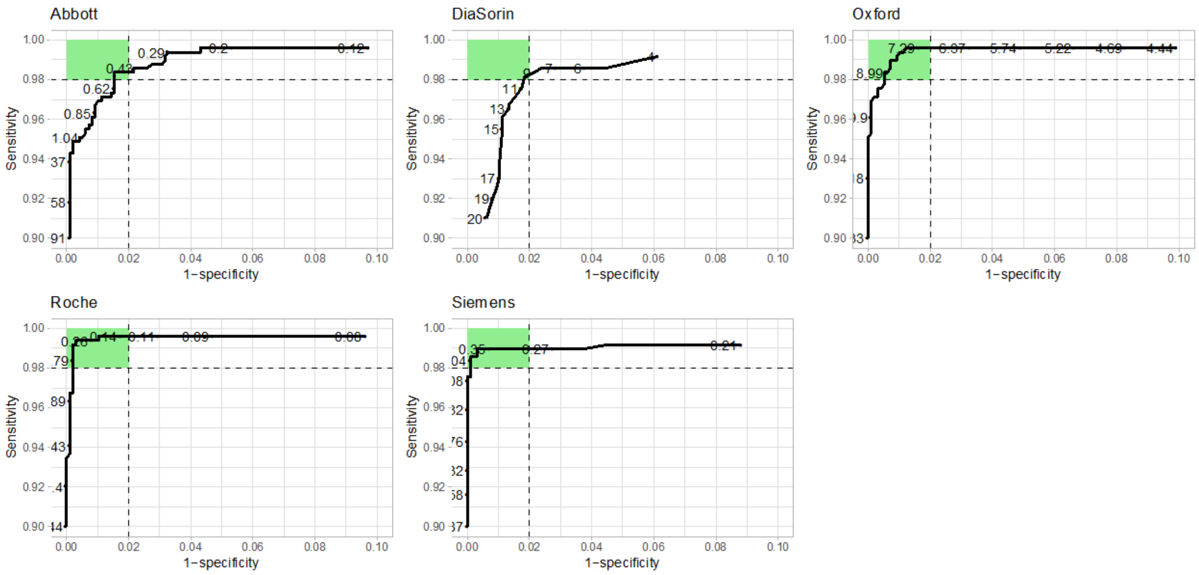

**Figure S8. Sensitivity and specificity (95% confidence intervals) plotted for each assay with alternative assay thresholds to keep specificity ≥98% and revised criteria to show samples ≥30 days after the appearance of first symptoms.** For each assay the lowest threshold that kept specificity ≥98% was chosen (Abbott=0·49, DiaSorin=10, OIA=7·19 million normalised standard units, Roche=0·128, Siemens=0·29). The UK MHRA target performance is shown (dashed line) including the required lower bound of the 95% confidence interval (dotted line) for both sensitivity and specificity. Data are presented for 976 known negative samples and 490 known positive samples run on each assay.

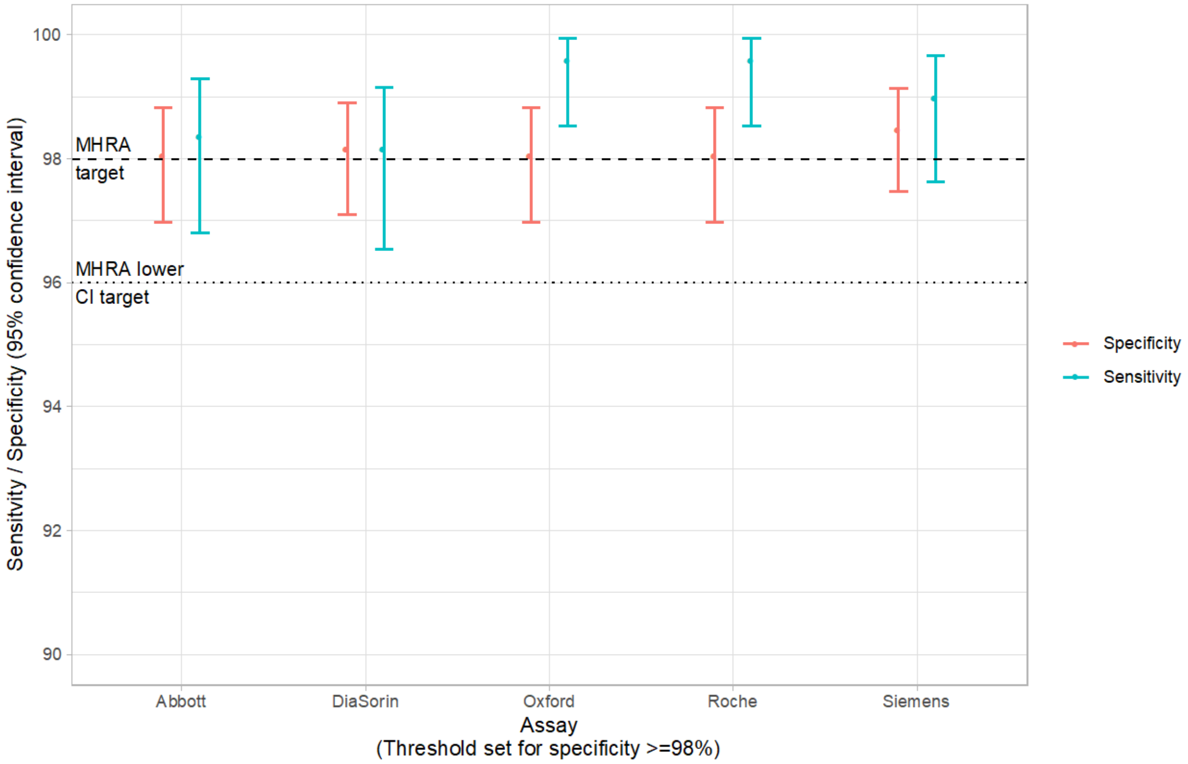

**Figure S9. Values for 8-point 1:2 dilution series of high-volume plasma controls defined as having high, medium and low titre antibodies (by EUROIMMUN; ratio values of 33·33, 4·34 and 2·50 respectively, where ratio is the optical density of the sample divided by the optical density of the calibrator [ratio of ≥1.1 is positive]; t**he S1 component of the SARS-CoV-2 spike protein is the antigen target for the EUROIMMUN assay**), and a known pre-pandemic negative control.** The "high" dilution series is represented by samples QC1001-QC1008, the "medium" dilution series by QC1009-1016, and the "low" dilution series by samples QC1017-1024. QC1025 is the negative control (sample BD01). Values were log(2) transformed prior to plotting.

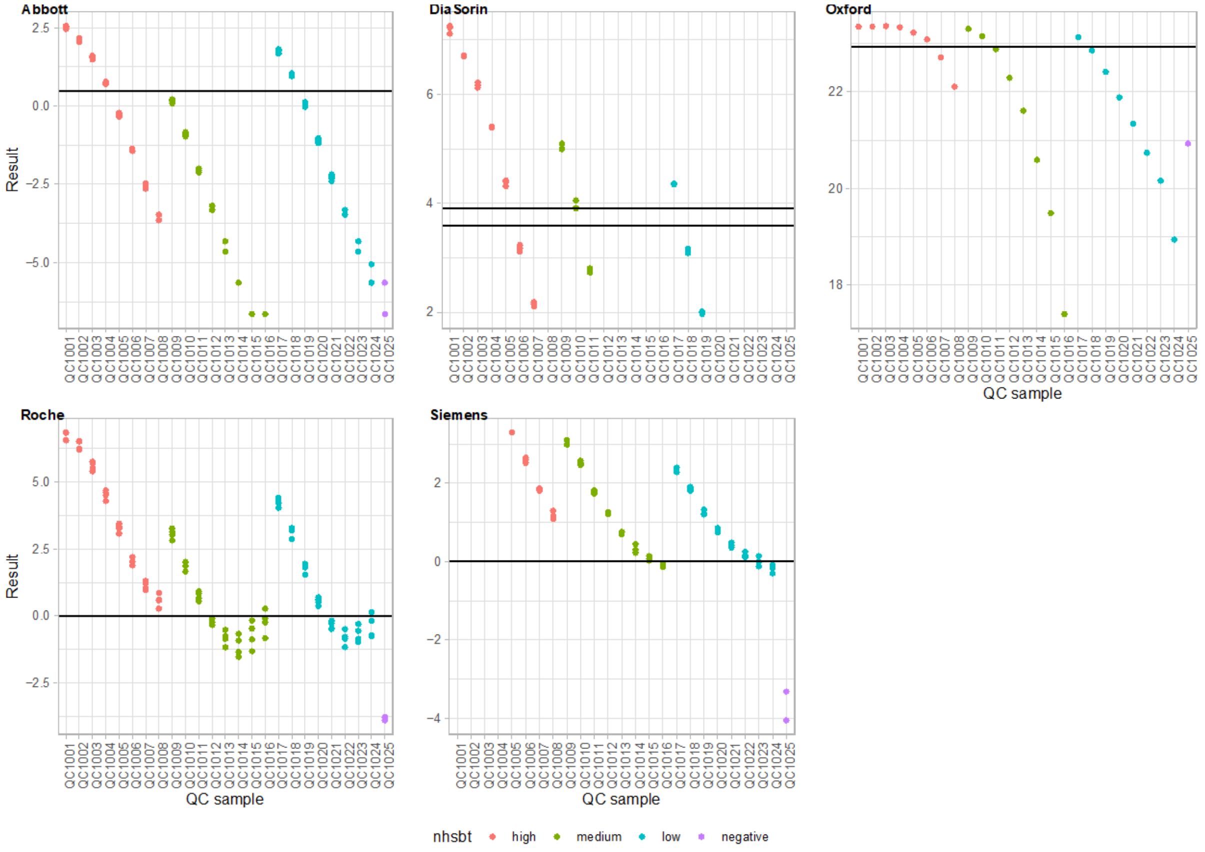

**Figure S10. Percentage of tests from SARS-CoV-2 RT-PCR-positive individuals positive over time by serology platform.** Samples from <20 days from symptom onset, excluded from the main analysis, are included here. Panel A shows the percentage by time since symptom onset and panel B the percentage by the time since the individual's first positive RT-PCR test.

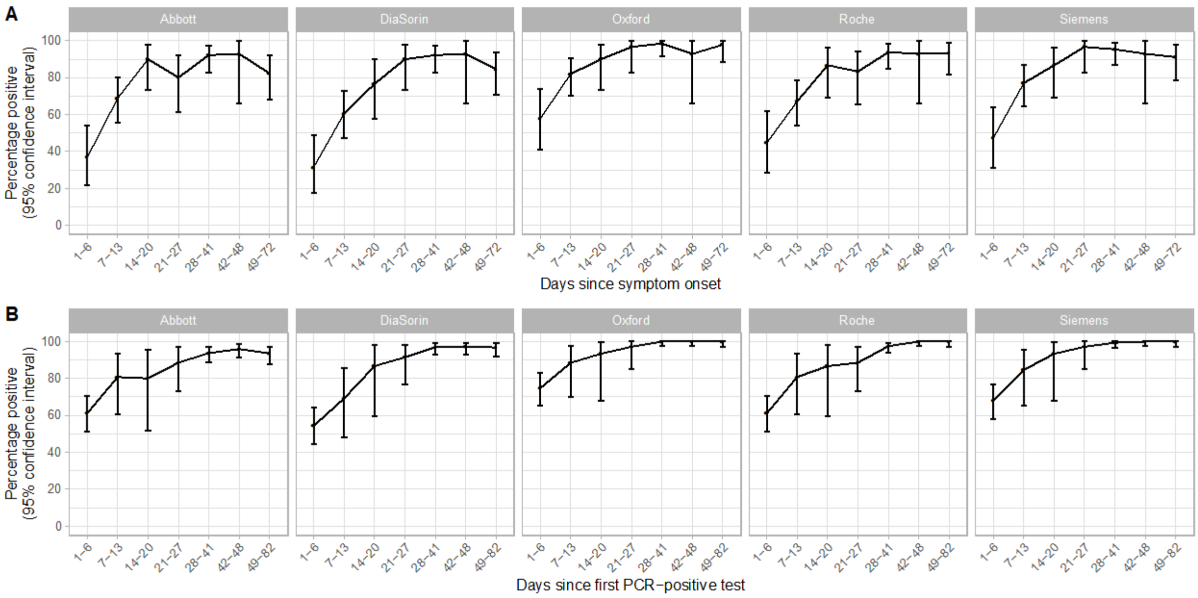

**Figure S11. Modelled antibody trajectories by assay, by day post-symptom onset.** First sample per patient only were included. The black line shows the modelled mean and the grey shading the 95% confidence interval for the mean.

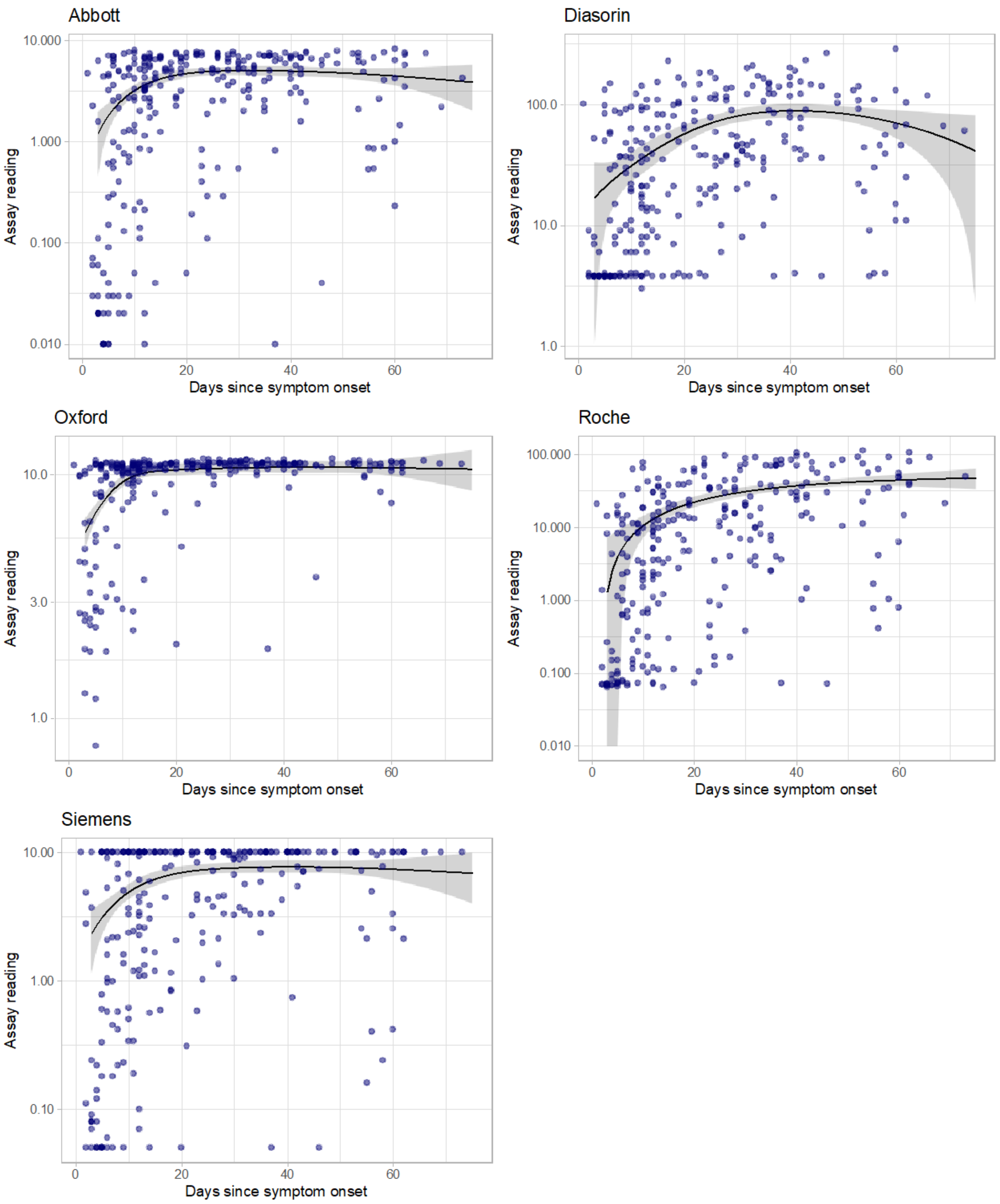

**Figure S12. Sensitivity for each assay by disease severity (asymptomatic, mild, severe, critical/death; n=158).** Disease severity was defined in line with WHO guidance(2) as follows: asymptomatic = no symptoms; mild = no oxygen requirement; severe = SaO2 ≤93%; critical = respiratory failure requiring intubation. Severity category was assigned on the day of sampling (asymptomatic n=13, mild n=122, severe n=16, critical/death n=7).

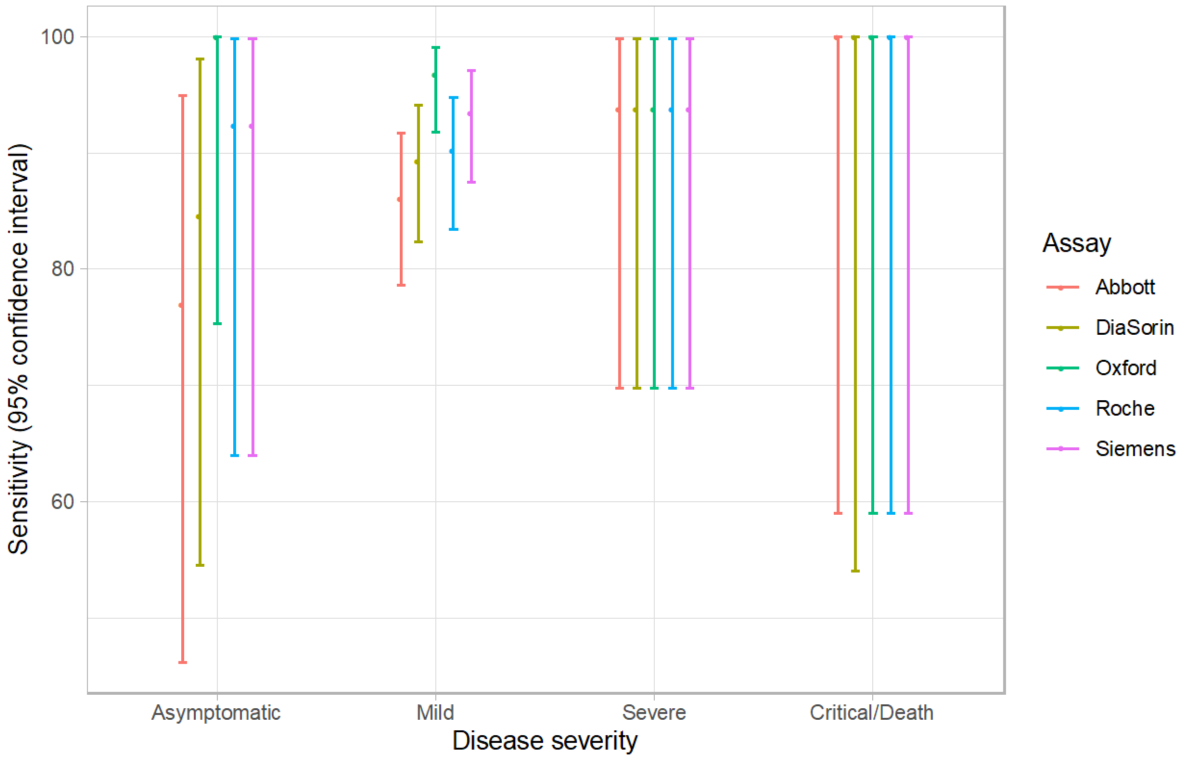

STARD checklist for evaluations of diagnostic accuracy

| Section & Topic | No | Item | Reported on page # |
|---|---|---|---|
| **TITLE OR ABSTRACT** | | | |
| | 1 | Identification as a study of diagnostic accuracy using at least one measure of accuracy (such as sensitivity, specificity, predictive values, or AUC) | Title, Abstract |
| **ABSTRACT** | | | |
| | 2 | Structured summary of study design, methods, results, and conclusions (for specific guidance, see STARD for Abstracts) | Abstract |
| **INTRODUCTION** | | | |
| | 3 | Scientific and clinical background, including the intended use and clinical role of the index test | Introduction |
| | 4 | Study objectives and hypotheses | Introduction |
| **METHODS** | | | |
| *Study design* | 5 | Whether data collection was planned before the index test and reference standard were performed (prospective study) or after (retrospective study) | Methods - study design |
| *Participants* | 6 | Eligibility criteria | Methods - study design |
| | 7 | On what basis potentially eligible participants were identified (such as symptoms, results from previous tests, inclusion in registry) | Methods - study design |
| | 8 | Where and when potentially eligible participants were identified (setting, location and dates) | Methods - study design, Appendix - Table S1 |
| | 9 | Whether participants formed a consecutive, random or convenience series | Methods - study design |
| *Test methods* | 10a | Index test, in sufficient detail to allow replication | Methods - study design and procedures, Appendix - supplementary methods section C, previous publication |
| | 10b | Reference standard, in sufficient detail to allow replication | Methods - study design |
| | 11 | Rationale for choosing the reference standard (if alternatives exist) | Methods - study design |
| | 12a | Definition of and rationale for test positivity cut-offs or result categories of the index test, distinguishing pre-specified from exploratory | Methods - study design, Appendix - Table S1, Appendix - supplementary methods section C |
| | 12b | Definition of and rationale for test positivity cut-offs or result categories of the reference standard, distinguishing pre-specified from exploratory | Methods - study design, Appendix - Table S1, Appendix - supplementary methods section C |
| | 13a | Whether clinical information and reference standard results were available to the performers/readers of the index test | Methods - procedures |
| | 13b | Whether clinical information and index test results were available to the assessors of the reference standard | Methods - procedures |
| *Analysis* | 14 | Methods for estimating or comparing measures of diagnostic accuracy | Methods - study design, procedures, outcomes and statistical analysis |

| | | | |
|---|---|---|---|
| | **15** | How indeterminate index test or reference standard results were handled | Methods - study design, procedures, outcomes and statistical analysis |
| | **16** | How missing data on the index test and reference standard were handled | Methods - study design, procedures, outcomes and statistical analysis, Appendix Figure S1 |
| | **17** | Any analyses of variability in diagnostic accuracy, distinguishing pre-specified from exploratory | Methods - study design, procedures, outcomes and statistical analysis |
| | **18** | Intended sample size and how it was determined | Methods - outcomes and statistical analysis |
| **RESULTS** | | | |
| *Participants* | **19** | Flow of participants, using a diagram | Appendix Figure S1 |
| | **20** | Baseline demographic and clinical characteristics of participants | Appendix Table S1 |
| | **21a** | Distribution of severity of disease in those with the target condition | Appendix Fig S12, dataset available at https://doi.org/10.6084/m9.figshare.c.5046032.v1 |
| | **21b** | Distribution of alternative diagnoses in those without the target condition | No detail available, mentioned in Discussion |
| | **22** | Time interval and any clinical interventions between index test and reference standard | Methods - study design, Appendix Table S1 |
| *Test results* | **23** | Cross tabulation of the index test results (or their distribution)<br>by the results of the reference standard | Table 1 |
| | **24** | Estimates of diagnostic accuracy and their precision (such as 95% confidence intervals) | Results, Table 1, Fig.1, Fig. 2, Appendix Table S5-S7, Fig. S2-S8, S10, S12 |
| | **25** | Any adverse events from performing the index test or the reference standard | Not applicable |
| **DISCUSSION** | | | |
| | **26** | Study limitations, including sources of potential bias, statistical uncertainty, and generalisability | Discussion |
| | **27** | Implications for practice, including the intended use and clinical role of the index test | Abstract, Discussion |
| **OTHER INFORMATION** | | | |
| | **28** | Registration number and name of registry | Not applicable |
| | **29** | Where the full study protocol can be accessed | https://doi.org/10.6084/m9.figshare.c.5046032.v1 |
| | **30** | Sources of funding and other support; role of funders | Abstract, Funding section |

## PRISMA checklist for systematic review

### PRISMA 2009 Checklist

| Section/topic | # | Checklist item | Reported on page # |
|---|---|---|---|
| **TITLE** | | | |
| Title | 1 | Identify the report as a systematic review, meta-analysis, or both. | Abstract, Research in context |
| **ABSTRACT** | | | |
| Structured summary | 2 | Provide a structured summary including, as applicable: background; objectives; data sources; study eligibility criteria, participants, and interventions; study appraisal and synthesis methods; results; limitations; conclusions and implications of key findings; systematic review registration number. | Abstract, Research in context, Appendix Supplementary Methods section A |
| **INTRODUCTION** | | | |
| Rationale | 3 | Describe the rationale for the review in the context of what is already known. | Research in context |
| Objectives | 4 | Provide an explicit statement of questions being addressed with reference to participants, interventions, comparisons, outcomes, and study design (PICOS). | Appendix Supplementary Methods section A |
| **METHODS** | | | |
| Protocol and registration | 5 | Indicate if a review protocol exists, if and where it can be accessed (e.g., Web address), and, if available, provide registration information including registration number. | No protocol registered for systematic review |
| Eligibility criteria | 6 | Specify study characteristics (e.g., PICOS, length of follow-up) and report characteristics (e.g., years considered, language, publication status) used as criteria for eligibility, giving rationale. | Supplementary Methods section A |
| Information sources | 7 | Describe all information sources (e.g., databases with dates of coverage, contact with study authors to identify additional studies) in the search and date last searched. | Research in context, Supplementary Methods section A |
| Search | 8 | Present full electronic search strategy for at least one database, including any limits used, such that it could be repeated. | Research in context, Supplementary Methods section A |
| Study selection | 9 | State the process for selecting studies (i.e., screening, eligibility, included in systematic review, and, if applicable, included in the meta-analysis). | Supplementary Methods section A |
| Data collection process | 10 | Describe method of data extraction from reports (e.g., piloted forms, independently, in duplicate) and any processes for obtaining and confirming data from investigators. | Supplementary Methods section A |
| Data items | 11 | List and define all variables for which data were sought (e.g., PICOS, funding sources) and any assumptions and simplifications made. | Supplementary Methods section A |
| Risk of bias in individual studies | 12 | Describe methods used for assessing risk of bias of individual studies (including specification of whether this was done at the study or outcome level), and how this information is to be used in any data synthesis. | Not evaluated |
| Summary measures | 13 | State the principal summary measures (e.g., risk ratio, difference in means). | Supplementary Methods section A |
| Synthesis of results | 14 | Describe the methods of handling data and combining results of studies, if done, including measures of consistency (e.g., $I^2$) for each meta-analysis. | Not applicable |



| Section/topic | # | Checklist item | Reported on page # |
|---|---|---|---|
| Risk of bias across studies | 15 | Specify any assessment of risk of bias that may affect the cumulative evidence (e.g., publication bias, selective reporting within studies). | Not evaluated |
| Additional analyses | 16 | Describe methods of additional analyses (e.g., sensitivity or subgroup analyses, meta-regression), if done, indicating which were pre-specified. | Not applicable |
| **RESULTS** | | | |
| Study selection | 17 | Give numbers of studies screened, assessed for eligibility, and included in the review, with reasons for exclusions at each stage, ideally with a flow diagram. | Research in context |

| | | | |
|---|---|---|---|
| | | | Supplementary Methods section A |
| Study characteristics | 18 | For each study, present characteristics for which data were extracted (e.g., study size, PICOS, follow-up period) and provide the citations. | Appendix Supplementary Tables S3, S4 |
| Risk of bias within studies | 19 | Present data on risk of bias of each study and, if available, any outcome level assessment (see item 12). | Not evaluated |
| Results of individual studies | 20 | For all outcomes considered (benefits or harms), present, for each study: (a) simple summary data for each intervention group (b) effect estimates and confidence intervals, ideally with a forest plot. | Not applicable |
| Synthesis of results | 21 | Present results of each meta-analysis done, including confidence intervals and measures of consistency. | Not applicable |
| Risk of bias across studies | 22 | Present results of any assessment of risk of bias across studies (see Item 15). | Not evaluated |
| Additional analysis | 23 | Give results of additional analyses, if done (e.g., sensitivity or subgroup analyses, meta-regression [see Item 16]). | Not applicable |
| **DISCUSSION** | | | |
| Summary of evidence | 24 | Summarize the main findings including the strength of evidence for each main outcome; consider their relevance to key groups (e.g., healthcare providers, users, and policy makers). | Research in context |
| Limitations | 25 | Discuss limitations at study and outcome level (e.g., risk of bias), and at review-level (e.g., incomplete retrieval of identified research, reporting bias). | Supplementary Tables S3, S4 |
| Conclusions | 26 | Provide a general interpretation of the results in the context of other evidence, and implications for future research. | Research in Context section |
| **FUNDING** | | | |
| Funding | 27 | Describe sources of funding for the systematic review and other support (e.g., supply of data); role of funders for the systematic review. | Funding section in Abstract and at end of manuscript |

**Supplementary References**


1. Emmenegger M, de Cecco E, Lamparter D, et al. Early plateau of SARS-CoV-2 seroprevalence identified by tripartite immunoassay in a large population. *medRxiv* 2020. DOI: https://doi.org/10.1101/2020.05.31.20118554.
2. Word Health Organisation (WHO). Report of the WHO-China Joint Mission on Coronavirus Disease 2019 (COVID-19). 2020. https://www.who.int/docs/default-source/coronaviruse/who-china-joint-mission-on-covid-19-final-report.pdf. Accessed: 18/Jun/2020.
